# Distinct Rhythmic Competencies Identified via Internet‐Based Assessment

**DOI:** 10.1111/nyas.70372

**Published:** 2026-08-11

**Authors:** Anna Fiveash, Nicholas E. V. Foster, Reyna L. Gordon, Simone Dalla Bella, Barbara Tillmann

**Affiliations:** ^1^ Université Bourgogne Europe, CNRS, LEAD, UMR5022 Dijon France; ^2^ Université Claude Bernard Lyon 1, INSERM, CNRS, Centre de Recherche en Neurosciences de Lyon CRNL U1028 UMR5292 Bron France; ^3^ The MARCS Institute for Brain, Behaviour and Development Western Sydney University Sydney Australia; ^4^ International Laboratory for Brain, Music, and Sound Research (BRAMS) Montreal Canada; ^5^ Department of Psychology University of Montreal Montreal Canada; ^6^ Centre for Research on Brain Language and Music (CRBLM) Montreal Canada; ^7^ Department of Otolaryngology‐Head & Neck Surgery Vanderbilt University Medical Center Nashville Tennessee USA; ^8^ Department of Hearing and Speech Sciences, VUMC Vanderbilt Brain Institute Vanderbilt University Nashville Tennessee USA; ^9^ VIZJA University Warsaw Poland

**Keywords:** beat, memory, music, perception, production, rhythm, rhythmic competencies

## Abstract

Rhythm is a fundamental element of music that recruits perception and production pathways in the brain, and occurs across different timescales. Research suggests that rhythmic processing is composed of several related, yet potentially distinct competencies, including perception, production, beat‐based processing, and sequence memory‐based processing. Based on a recent lab‐based study by Fiveash et al., the current experiment investigated whether these distinctions could be replicated with more participants (*n* = 300) completing an internet‐based protocol, with the potential to be scaled up to larger samples. Production tasks included unpaced tapping and paced tapping to music (from the Battery for the Assessment of Auditory Sensorimotor and Timing Abilities). Perception tasks included the beat alignment test, the Burgundy best Musical Aptitude Test (synchronization), and the beat‐based advantage task. Principal component analyses revealed strong overlap across tests. However, separations were shown between perception and production tasks, and unpaced and paced tapping tasks. Cluster analyses revealed participants with different rhythmic profiles, for example, (1) strong beat‐based rhythm perception, weak sequence memory‐based rhythm perception; and (2) strong production, weak perception. These results have implications for future research investigating individual differences in general and clinical populations, with the potential to guide clinical interventions.

## Graphical abstract

A set of rhythm tasks administered via the internet showed evidence for distinct rhythmic competencies relating to perception versus production as well as sequence memory‐based perception compared to beat‐based perception. These findings replicate lab‐based results. Distinct clusters of participants were found, resulting in the identification of different rhythmic profiles.

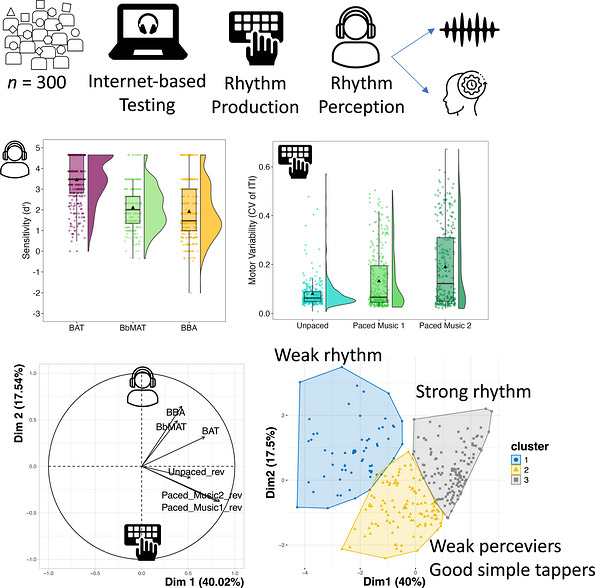

## 1 Introduction

Imagine a pop concert—the musicians are playing different instruments that are perfectly synchronized together by following an underlying beat (i.e., the pulse, underscored by the drums). They are watching the audience dancing to the music—and most people are moving along in time with the beat. This situation is quite common; and it includes several related and potentially separable subprocesses involved in perceiving and producing rhythm [1]. There are also a large range of interindividual differences displayed in the capacity to move along in time with the music, or to perceive if one member of the audience is moving out of time. Research has shown that such rhythmic competencies may be partially separable, especially in relation to perception compared to production tasks [2, 3, 4], and tasks requiring beat‐based processing compared to those requiring sequence‐based memory [5, 6, 7].

In terms of perception compared to production, case studies have revealed participants who are beat‐deaf, showing impairments in beat perception (i.e., estimating if a metronome is aligned or not to the beat of short musical excerpts, detecting irregularity in a regular sequence), but not in beat production, with normal scores when tapping along to the beat of music or a metronome [2]. The reverse has also been found—participants who performed poorly when tapping along to a rhythm, but were at the same level as controls for perception tasks [4]. Interestingly, some beat‐deaf participants [8] (*n* = 2) also had trouble synchronizing to an external beat (perhaps related to their perceptual/beat‐finding impairment), and adapting to changes in timing (see also Ref. [9]). However, they did not differ from controls in variability on a spontaneous (unpaced) tapping task, suggesting a potential distinction between unpaced and paced tapping tasks. Although findings from case studies should be interpreted with caution because of their limited generalizability and the inferential constraints inherent to single‐case designs, similar patterns of results are also reflected in larger datasets showing that perception and production tasks do not always correlate [10, 11, 12]. Such dissociations and lack of correlations demonstrate the complexity of rhythmic skills, and suggest that different individuals may show different patterns of rhythmic competencies [3, 13].

Distinctions have also been shown between beat‐based rhythm tasks, such as detecting if an external pulse is aligned to the beat of music, and sequence memory‐based rhythm tasks, such as judging if rhythms are the same or different, or reproducing beat‐based rhythms from memory [5, 7]. For example, Refs. [7, 14] showed a distinction between production tasks involving beat‐based processing (tapping to a metronome, adjusting to tempo changes of the metronome while tapping) and memory‐based processing (learning and then drumming along to rhythmic sequences, remembering temporal patterns and repeating them). It was further shown that performance on these tasks correlated with subcortical (reflecting shorter timescales) and cortical (reflecting longer time scales) frequency‐following responses, respectively [14]. Such distinct rhythmic competencies are likely to relate to more general cognitive capacities, especially working memory capacity (WMC) in the case of sequence memory‐based tasks. For example, a recent study showed that WMC contributes to performance on a memory‐based rhythm perception task, but not a beat‐based rhythm perception task in children [6]. Further, it has been shown that WMC correlates with complex rhythm discrimination in adults who stutter and controls, but only correlates with simple rhythm discrimination in adults who stutter [15]. These differences might relate to different underlying mechanisms supporting beat‐based and memory‐based expectations [16].

Understanding patterns of rhythmic competencies in the general population is a critical first step to inform further systematic investigation of rhythmic impairments in clinical populations [17, 18, 19], and their links with different cognitive functions [20, 21, 22]. Research has started to suggest a link between atypical rhythm processing and developmental speech and language disorders [23, 24, 25, 26, 27, 28]. For example, poorer rhythm skills (compared to controls) have been shown in children with dyslexia [29, 30, 31, 32, 33] (see also Ref. [34] for a larger study investigating genetic links between rhythm and dyslexia), developmental language disorder [35, 36], stuttering [15, 37, 38, 39], attention‐deficit/hyperactivity disorder (ADHD) [21, 40, 41, 42], and developmental coordination disorder [43, 44, 45]. However, different rhythm tests are used across different studies, so it can be challenging to tease apart whether distinct rhythmic competencies are linked with specific language‐related impairments or whether they function as transdiagnostic features contributing to diverse clinical presentations within a given disorder [26, 28]. Further, clinical populations can be difficult to recruit, so an internet‐based platform would be particularly valuable to access such populations and make the assessment more inclusive by reducing barriers associated with in‐person testing, such as travel, mobility limitations, fatigue, and scheduling constraints.

There are also large individual differences in rhythm skills within general and clinical populations, which appear to relate to other cognitive skills, and might differ based on the rhythm task being measured. Although the majority of participants can synchronize to an external beat, 14% of 100 typical undergraduate students showed difficulty synchronizing [46]. Further, large individual differences have been shown in relation to spontaneous motor tempo, which differs substantially across studies, as well as in relation to both intrinsic (i.e., age, personality) and extrinsic (i.e., time of day) factors [47]. Further, depending on whether a tapping task was paced or unpaced, different patterns of correlation were observed with measures of attention, auditory temporal precision (backward masking task), and reading [22], suggesting distinctions across rhythm tasks. The link between rhythm and executive functions has also been shown [21]—when children and adults with ADHD were separated into good and poor beat trackers: the good beat trackers performed better on inhibition and flexibility executive function tasks compared to the poor beat trackers. This research aligns with work showing that rhythmic (musical) training enhances executive functions [48, 49, 50], suggesting that these skills may be related and can be improved with training.

Finally, a recent study [13] tested 79 participants on the full Battery for the Assessment of Auditory Sensorimotor and Timing Abilities (BAASTA) battery, which measures several rhythm perception and production competencies. Machine learning and graph theory were used to reduce the dimensionality of the perception and production tasks to just two perceptual and two motor tasks (and their interactions), which were able to differentiate musicians compared to nonmusicians. Interestingly, the interaction between perception and production variables was stronger for musicians compared to nonmusicians, suggesting also an interest of combining different rhythm tasks.

Based on these different patterns of results revealed in the literature, our previous, lab‐based study (*n* = 31) [3] was run to shed light on individual differences in rhythmic competencies across multiple rhythm tasks. In this previous study, participants performed five rhythm perception tasks and four rhythm production tasks, aiming to cover several different rhythmic competencies with tests from different labs. The results showed clear distinctions between participants’ performance in the perception and production tasks. They also showed different patterns of performance for tasks involving beat‐based rhythm perception compared to sequence memory‐based rhythm perception. Further, distinct clusters of participants emerged who showed selectively strong or weak performance across different tasks, suggesting different combinations of rhythmic competencies across participants.

The current study aimed to replicate and extend this lab‐based study using a larger, internet‐based sample of 300 participants. Replication using a larger sample via internet‐based testing can pave the way to scaling up the approach to test larger participant groups, as well as difficult‐to‐reach populations, such as specific clinical populations with limited participant pools. We were also interested to see whether the results from Fiveash et al. [3] could be replicated with an internet‐based sample, where additional variability (both measurement and stimulus presentation timing) and less control would be expected compared to lab‐based settings. Internet‐based testing has increased substantially since the COVID‐19 pandemic, with several testing tools available [51]. However, issues with data quality remain, including those related to latency and jitter for timing‐sensitive measures [52], and participant's behavior during the testing linked to unsupervised performance [51]. To adapt the lab‐based testing from Fiveash et al. [3] to internet‐based testing, we (a) included a subset of perception and production tasks to reduce testing time to approximately 35 min; (b) included checks of attention across three different tasks, where participants were given trials with an obvious answer, or were instructed to answer in a certain way; (c) recruited through a reputable participant platform, Prolific; and (d) paid the participants appropriately. Further, we did not calculate phase synchronization metrics, which would be influenced by latencies with audio presentation across computers [52], but calculated within‐participant tapping variability.

It is hypothesized that the principal component analyses (PCAs) will reveal distinct dimensions of performance for perception compared to production tasks, as well as between beat‐based perception and sequence memory‐based perception tasks. It is also hypothesized that clusters of participants will emerge with distinct patterns of performance based on these theoretically motivated distinctions (perception vs. production tasks; beat‐based vs. sequence memory‐based tasks). Although a distinction between unpaced and paced tapping tasks did not emerge in Fiveash et al. [3], it is possible that this distinction will emerge in this larger sample, reflecting synchronization to an external beat compared to internal generation of a beat (as in Refs. [8, 22]).

## 2 Materials and Methods

### 2.1 Participants

Three‐hundred participants were recruited online through Prolific (108 female, 190 male, two prefer not to answer; *M*_age_ = 36.52, *SD* = 11.93, range = 18–74). All participants rated English as their first (*n* = 294) or joint first (*n* = 6) language, with 201 monolingual and 99 multilingual participants. Three participants indicated hearing problems, but none reported trouble hearing the stimuli in the experiment. A range of education was reported: 74 completed high school or equivalent; 45 completed some college or associate's degree; 144 completed a bachelor or equivalent; 29 completed a master's degree; and eight did not respond. Eighty‐one participants reported formal music training (*M* = 4.59 years, *SD* = 4.34, Med = 3 years, range = 1–25) and 22 participants reported formal dance training (*M* = 6.27 years, *SD* = 4.48, Med = 5.50 years, range = 1–14). This study was approved by the Comité d’éthique de la recherche en éducation et psychologie (CEREP) of the Université de Montréal (#2023‐4944), and participants were informed about the nature of the experiment and gave their consent before the experiment.

### 2.2 Online Setting

The experiment was distributed to eligible participants through Prolific, using the standard sample (i.e., distributed to all available participants), with screening criteria that their first language was English (to fully understand the instructions, and have a more homogenous sample). Participants were told to complete the experiment on a computer and with headphones or speakers. Participants responded that they completed the experiment on: a laptop (*n* = 196), a desktop computer (*n* = 102), or a mobile device (*n* = 2). A built‐in keyboard was used for 196 participants, a wired keyboard for 73, a wireless keyboard for 25, a wired mouse for four, and a touch screen for two. For the method of listening, 132 reported wired headphones, 89 reported using speakers, 54 reported wired in ear headphones, 24 reported wired earbud headphones, and one reported wireless earbud headphones. Considering the variability related to online testing [53], especially using the spacebar as a tapping measure [52], only measures of tapping variability were included in the analysis, rather than measures of phase‐locking. However, it should be noted that a study across several operating systems, keyboard types, and software showed that internet‐based testing has reasonable accuracy and precision in terms of keyboard‐based responses [51].

### 2.3 Tasks

Due to time limitations with internet‐based testing, a subset of two production and three perception tasks from Fiveash et al. [3] that emerged as the most distinctive were selected to be included in the current study, with the goal to capture different rhythmic competencies that emerged in the original study and in the literature. The production tasks were unpaced tapping and paced tapping to music taken from the BAASTA [10, 11]. For both tapping tasks, participants were asked to tap their computer spacebar with the index finger of their dominant hand. There was no acoustic feedback provided. The unpaced tapping task also provides a measure of spontaneous motor tempo [54], which is reported below for completeness. The perception tasks were the beat alignment test (BAT; originally from Iversen and Patel [55], stimuli from Dalla Bella et al. [10]); the Burgundy best Musical Aptitude Test (BbMAT) synchronization task (from http://leadserv.u‐bourgogne.fr/~cimus); and the beat‐based advantage (BBA) task (stimuli adapted from Gordon et al. [56], originally adapted from Grahn and Brett [57]). The implementations and stimuli of these tasks were the same as those reported in Fiveash et al. [3], adapted to internet‐based testing.

#### 2.3.1 Unpaced Tapping

In the unpaced tapping task, participants were asked to tap as regularly as they could, at a comfortable and consistent speed. A green square appeared on the screen with the word “TAP!” to indicate that participants should start tapping. While they were tapping, the words “Keep tapping” were on the square. Participants were told to stop tapping after 60 s.

#### 2.3.2 Paced Tapping to Music

In the paced tapping task, participants were asked to tap along to the regular beat of music, defined as the sense of a regular pulse in the music to which participants might clap or tap their foot. The specific tapping rate was not indicated; participants tapped at the rate (metrical level) corresponding to their perception of the beat. Participants were instructed to only move their finger, not the rest of their body, and they were reminded to tap in synchrony with the beat, while keeping the same time interval between the taps. A green square appeared on the screen with the word “TAP!” to indicate that participants should start tapping. While they were tapping, the words “Keep tapping” were on the square. Participants had two practice trials and could repeat the instructions and practice trials if they did not understand. Two pieces of music (from the BAASTA, Dalla Bella et al. [11]) were presented twice, resulting in four trials of 38 s each, corresponding to 64 beats. Paced Music 1 was the *Badinerie* (Bach) and Paced Music 2 was the *William Tell Overture* (Rossini). Both had a 600 ms inter‐onset interval with a 4/4 time signature, with a tempo of 100 bpm. The task took approximately 4 min.

#### 2.3.3 Beat Alignment Test

In the BAT, participants were asked to judge whether a woodblock sound was aligned or not aligned with the beat of the musical excerpts. The musical excerpts were the same as those used in the paced tapping task and in Fiveash et al. [3], which were taken from the BAASTA [11]. Participants were given two examples in the aligned condition, and two examples in the not aligned condition (one period, one phase error). They then completed four practice trials (one aligned and three not aligned trials) with feedback. Participants could repeat the example and practice trials if needed. Across the 24 trials of the experiment, there were eight trials on the beat, eight with a period error (four 10% faster and four 10% slower than the beat), and eight with a phase error (i.e., four shifted before, four shifted after the beat by 33%). Participants had to wait until the end of the music (11–12 s) to respond. This task took approximately 8 min.

#### 2.3.4 Burgundy best Musical Aptitude Test Synchronization Task

In the BbMAT synchronization task (developed by Emmanuel Bigand and Philippe Lalitte), participants were told that they would hear rhythmic excerpts played by several musicians with percussion instruments, and that these musicians would either play well together (i.e., be synchronized) or not play well together (i.e., be unsynchronized). Participants were asked to assess whether the musicians were synchronized with each other or not. They were asked not to move to the music, just to listen carefully. They first heard one synchronized and one unsynchronized example (that they could repeat), followed by 16 experimental trials (half synchronized, half unsynchronized). Stimuli were the same as in Fiveash et al. [3], with a duration of 17 s (see example here: https://osf.io/4r5hd/overview?view_only=58f4733b79a84624928edd59c25d6ef0). Rhythms were in the style of the Brazilian batucada, containing a polyrhythmic structure with syncopations, with four instrument streams. To create the unsynchronized versions, three of the four streams had their onsets randomly shifted by different values between 30 and 60 ms (see Fiveash et al. [3] for more details). This subtle manipulation made the task more complex than the judgment of the external beat alignment in the BAT. Stimuli were presented in a randomized order fixed for all participants, where the same item was not repeated in a row in its synchronized and unsynchronized version, and no more than three of the same trial type were repeated in a row. Participants could only respond at the end of the stimulus; the task took approximately 6 min.

#### 2.3.5 Beat‐Based Advantage Task

In the BBA task, participants heard three short rhythmic patterns (separated by 1500 ms silence) and were asked to judge whether the third rhythm was the same or different from the first two identical rhythms. Participants were asked not to count or tap when listening to the rhythms and completed two practice trials with feedback. There were 16 trials in total (half same, half different). Eight trials were simple (with a strong beat), and eight trials were complex (with a weak beat). To help navigate participants, a schema with “Rhythm 1 → Rhythm 2 → Rhythm 3” was shown on the screen while they were listening to the rhythms. The rhythms were the same as those used in Fiveash et al. [3], using pure tones, which had been adapted from Gordon et al. [56], and were originally adapted from Grahn and Brett [57]. The three rhythms were 12–13 s long and the participant could only respond at the end of the trial. The task took about 6 min.

#### 2.3.6 Questionnaire

Finally, participants were asked basic demographic questions (age, gender identity, level of education, native language, different languages spoken, whether they had any hearing problems). They were also asked to report whether they had any formal music or dance training, including instrument/voice/dance style, years of training, and age started. They were then asked how much they agreed with four musical statements on a 7‐point Likert scale from “completely disagree” to “completely agree”. The first two aimed to assess subjective rhythmic skills: (1) “I can tap in time with a musical beat” (adapted from Ref. [58]) and (2) *“*I am a good dancer; I dance in rhythm with the music.” The last two aimed to detect potential amusia, which were taken from Ferreri and Rodriguez‐Fornells [59]: (3) *“*I have trouble recognizing songs without the lyrics” and (4) *“*I am rarely able to notice if someone is singing out of tune.” Note that these are somewhat similar to some of the statements in the Goldsmiths Musical Sophistication Index [60]; however, for time purposes, we selected only these four relevant ones. Additional speech and language questions were administered but will be reported in a separate article.

### 2.4 Attention Checks

Three attention checks were included to ensure that the internet‐based participants were paying attention during the tasks. (1) The BBA had one final additional trial that included two extremely dissimilar rhythms, and participants judged if they were the same or different; (2) The BbMAT had one final additional trial that included a rhythm with no regularity (an irregular rhythm from Fiveash et al. [61]), and participants were asked whether it was synchronized or not; (3) In the questionnaire, the second‐to‐last question asked participants to select “Antarctica” from the dropdown menu.

### 2.5 Procedure

After reading the information and consent screen, participants checked a box to give their consent to participate in the experiment. Participants were asked about the device and type of headphones they were using, and did a volume check. They then completed unpaced tapping, paced tapping to music, BAT, BbMAT, BBA, and the questionnaire in this order. The whole experiment took approximately 35 min, and participants were paid 5.25 pounds (9 pounds/h) in line with Prolific payment rates.

### 2.6 Screening

To check level of attention to the experiment, we recorded the amount of time throughout the full experiment that participants stayed on the experimental screen. On average, participants stayed on the experimental screen 99.07% of the time (SD = 4.17%, range = 59%–100%, median = 100%). Most participants stayed focused on the experimental screen for the duration of the experiment (*n* = 261). Of the 38 that left the screen at some stage during the experiment, the range was 59%–99.9% on the screen (*M* = 92.6%, *SD* = 9.56%), and only three of these participants failed attention checks (one for the BBA, one for the BbMAT, and one for the questionnaire). The median time spent completing the full study was 33 min, ranging between 25 min to 102 m. No participant failed all three attention checks or performed consistently poorly (i.e., more than 2–3 *SD* from the mean) across all tasks. In total, two participants failed the BBA check, eight participants failed the BbMAT check, and five participants failed the questionnaire check. One participant failed both the BbMAT and the questionnaire check, but not the BBA check. One participant had a *d* prime (*d′*) score more than 3 *SD* from the mean in the BBA, but performed well in the BAT and BbMAT tasks. There were no participants more than 3 *SD* from the mean performance in BAT or BbMAT. We thus decided to keep all 300 participants in the analysis. However, it should be noted that removing the 15 participants who either failed an attention check or had performance more than 3 *SD* from the mean performance in the perception tasks did not change the dimensions or clusters reported.

### 2.7 Analysis

#### 2.7.1 Perception and Production Tasks

For the perception tasks, *d*′ and response bias *c* were calculated as measures of sensitivity and bias, respectively, to detecting the not aligned (BAT), different (BBA), and unsynchronized (BbMAT) stimuli, in line with signal detection theory [62]. Hits and false alarms (FAs) were used to calculate *d′*, with the formula *z*(Hits) − *z*(FA). Extreme values of 0 and 1 were corrected to 0.01 and 0.99, respectively. Response bias *c* was calculated by the formula (*z*(Hits) + *z*(FA)) × 0.50, resulting in positive values reflecting a bias to respond as not aligned/different/unsynchronized, and negative values reflecting a bias to respond as aligned/same/synchronized (see Supporting Information Section 1 for response bias *c* results and descriptive statistics, as well as Tables S1, S2, and Figures S1–S3).

For the production data, preprocessing steps were conducted similarly to Dalla Bella et al. [11]. Spurious and initial taps were removed to reduce variability related to the onset of the tapping or stimulus. These included any taps less than 100 ms after the previous tap, the 10 initial taps, and for the paced tapping trials, any taps more than 0.5× interstimulus interval (ISI) before the 10th stimulus time (metronome or musical beat) or after the last stimulus time. Then, the intertap intervals (ITIs) were calculated, and outlier intervals were detected. Outliers were defined as in Dalla Bella et al. [11], that is, when the ITI was < Q1 – 3 × IQR or > Q3 + 3 × IQR, where Q1 is the first quartile of ITI values in the trial, Q3 is the third quartile, and IQR is Q3 – Q1. For each ITI outlier, that ITI value and the following one were removed for the ITI statistics. For the paced trials, for each ITI outlier, the tap event that ended that interval was excluded (for vector length *R*, see Supporting Information Section 1.2 and Figure S3). For the paced tapping to music trials, all of these steps were repeated for each of the metrical levels: 0.5× ISI, 1× ISI, and 2× ISI. The scores for the metrical level with the highest circular vector length were retained, as this effectively reflects the metrical level that best fits the participant's tapping. After cleaning, the coefficient of variation of the ITI (CV of ITI) was calculated as the *SD* of the ITI/mean ITI, reflecting motor variability. The CV of ITI was used in the following analyses.

#### 2.7.2 Principal Component and Hierarchical Cluster Analyses

Three PCAs were run, on (A) all *d′* values from the perception tasks, (B) all CV of ITI values from the production variables, and (C) all *d′* and CV of ITI values from perception and production tasks, respectively. A further PCA is reported in Supporting Information (Section 2.2 and Figure S6) with the production variables and responses to the two production statements “I can tap in time to a musical beat” and “I am a good dancer; I dance in rhythm with the music.” Clustering was performed using the hierarchical classification on principal components within the R package *FactoMineR* [63]. Considering that the CV of ITI was significantly more variable for paced tapping to Music 2 (PM2; *M* = 0.19, *SD* = 0.16), compared with paced tapping to Music 1 (PM1; *M* = 0.13, *SD* = 0.12), *t*(297) = 9.38, *p* < 0.001, we decided to enter PM1 and PM2 separately into the PCA (note that PM2 is consistently shown as more difficult than PM1 [3, 11]). This decision allows for more variation across participants, as well as including three perception and three production variables. Music 1 and Music 2 each had two trials that were averaged for more robust CV of ITI measures. In the PCA, the CV of ITI scores were reversed so that higher scores reflect better performance, to facilitate interpretation. Two participants were missing all tapping data, so they were excluded from analyses with production data, but were included in the perception‐only analyses.

Clusters were validated using ANOVAs and multinomial logistic regression. For ANOVAs, the package *ezanova* was used in the case that Levene's test was not significant, otherwise a Welch test (oneway.test) was used. Effect sizes in both cases are reported with omega squared (*omegasquared* function in the *effectsize* package in R), as it reduces bias related to unequal sample sizes [64, 65]. In the case of equal variances, *t*‐tests were run for contrasts (with Holm–Bonferroni correction for multiple comparisons), otherwise the Games–Howell test was run for unequal variances (with a Tukey adjustment for multiple comparisons). Cohen's *d* is reported for contrast effect sizes (small effect ≥ 0.2, medium effect ≥ 0.5, large effect ≥ 0.8; Cohen [66]). To investigate overlap between clusters and self‐reported years of musical training, Welch's one‐way ANOVAs were run to see whether years of musical training could be predicted by cluster membership, in each PCA.

Multinominal logistic regression was run using the *nnet* package [67] in RStudio, and then cross‐validated with a leave‐one‐out cross‐validation approach (LOOCV) using the *caret* package [68]. Using this approach, the model was trained on 299 (for perception) or 297 (for production) data points and then the nonincluded case was classified into a cluster based on its pattern of results (repeated for each participant). The classification accuracy shows whether the clusters emerging from the PCA represent meaningful and distinguishable clusters based on the raw task performance, acknowledging that there is some circularity in this measure, as the clusters were originally derived from the PCA analysis using the same raw data. Further information for output sensitivity, specificity, and precision for each model is presented in Table S4.

## 3 Results

### 3.1 Descriptive Results

**FIGURE 1:**
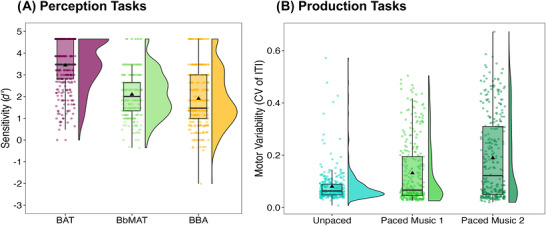
(A) *d*′ values for the perception tasks. (B) Motor variability for the production tasks (coefficient of variation of the intertap intervals, CV of ITI). Boxplots show the median as the horizontal black line, the mean as the black triangle, and the interquartile range as the box. The violin plot shows the density of responses, and each dot is an individual data point. The CV of ITI values reported here are the initial values before being reversed for inclusion in the analyses. BAT = beat alignment test; BBA = beat‐based advantage task; BbMAT = Burgundy best Musical Aptitude Test synchronization task.

Figure 1 shows the *d′* and CV of ITIs (i.e., showing motor variability) for the perception and production tasks. Please see Supporting Information Section 1 and Table S1 for the simple versus complex conditions in the BBA and the period versus phase conditions in the BAT, as well as the *d′* prime values, response bias *c*, accuracy, and hits‐FA rates for the perception tasks.

For the tapping tasks, CV of ITI values, vector length R, and ITIs are reported in Table S2 and shown in Figure S3. For the unpaced tapping task, the mean ITI was 516.1 ms (SD = 245.4, range = 156.4–2059.0 ms). Although the average ITI was faster than a previous online study [54] with over 3000 participants (*M* = 780 ms), the range was similar (123–2150 ms).

Compared to the lab‐based study of 31 participants in Fiveash et al. [3], there was no systematic benefit of lab‐based compared to internet‐based assessment. See Figure S4 and Supporting Information Section 1.3. Compared to the lab‐based study in Fiveash et al. [3], performance on the BAT was higher in the current sample, whereas performance on the BBA was lower. There was no difference for the BbMAT. For unpaced tapping, performance was more variable in the lab‐based study, and there was no difference between lab‐based and internet‐based performance for PM1 or PM2. Beyond potential influences of internet‐based versus lab‐based implementation, differences between the two studies might be due to differences in age, musical training, and educational background, as well as the larger sample in the current study.

### 3.2 Principal Component Analysis—Perception Tasks

**FIGURE 2:**
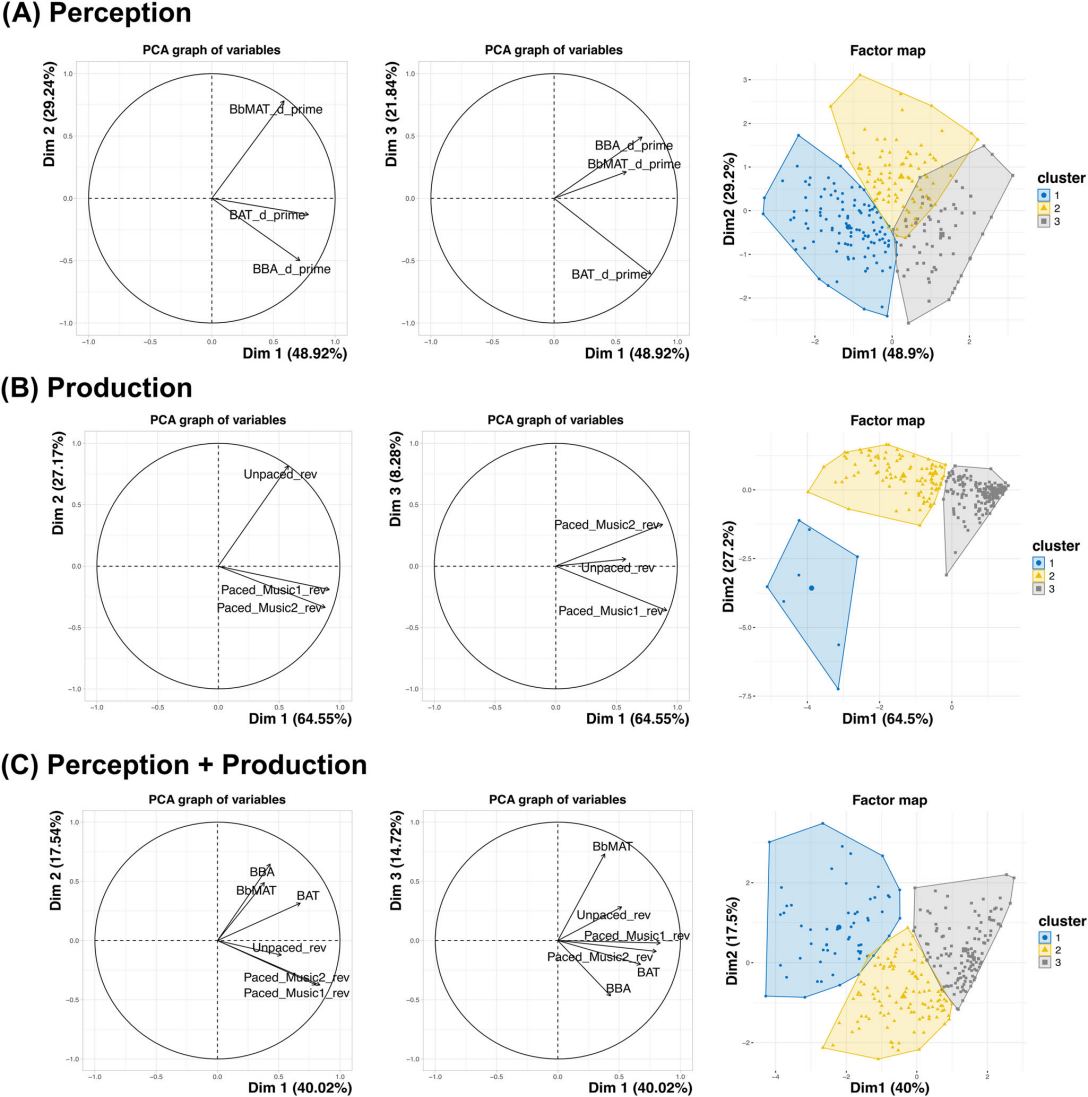
Dimensions and clusters emerging from the principal components analyses for (A) all perception variables, (B) all production variables, and (C) all variables. Paced tapping measures (Paced_Music1_rev, Paced_Music2_rev, Unpaced_rev) were reversed so that positive scores correspond to higher accuracy in tapping. BAT = beat alignment test; BBA = beat‐based advantage task; BbMAT = Burgundy best Musical Aptitude Test synchronization task.

The perception PCA revealed three components accounting for 100% of the variance (see Table 1 for all statistical information and Figure 2A for visualizations). Note that tests are presented in order of greatest contribution (positive contributions presented first). Dimension 1 positively correlated with performance on all tasks: BAT, BBA, and BbMAT, reflecting general rhythm perception performance. Dimension 2 positively correlated with BbMAT, and negatively with BBA and BAT. This dimension appears to primarily reveal a distinction between BbMAT and BBA performance, with BAT correlating more closely with BBA than BbMAT along this dimension. Dimension 2 could reflect a distinction between complex beat extraction (BbMAT) and sequence‐based memory (BBA). However, as BAT negatively correlated with Dimension 2, it could also reflect a distinction between more complex (i.e., BbMAT) versus less complex (i.e., BBA, BAT) rhythm tasks. BbMAT can be considered a more complex task, as the synchronization manipulation is quite subtle, within the same sound stream, and implemented over several instruments; whereas the stimuli used in the BBA (pure tones) and the BAT (external sound aligned with MIDI music) could be considered simpler. Dimension 3 positively correlated with BBA and BbMAT, and negatively correlated with BAT. This dimension appears to largely reveal a separation between BBA and BAT, with BbMAT correlating more closely to the BBA than BAT. This dimension could be interpreted as accounting for sequence‐based memory (based on the BBA), rather than beat alignment (based on the BAT). The complexity of the BbMAT stimuli may have engaged some memory processes, which could explain the positive correlations with the BBA and BbMAT.

**TABLE 1:** Overview of the dimensions, variance explained, contributing factors, and interpretation of the principal components analyses (PCA) for perception, and production.

PCA	Dim	Variance	Contributing tasks	Interpretation
Perception	Dim 1	48.92%	BAT: *r*(298) = 0.78, *p* < 0.001 BBA: *r*(298) = 0.71, *p* < 0.001 BbMAT: *r*(298) = 0.59, *p* < 0.001	General rhythm performance
	Dim 2	29.24%	BbMAT: *r*(298) = 0.78, *p* < 0.001 *BBA: *r*(298) = −0.50, *p* < 0.001 *BAT: *r*(298) = −0.13, *p* = 0.02	Complex beat extraction vs. sequence‐based memory
	Dim 3	21.84%	BBA: *r*(298) = 0.49, *p* < 0.001 BbMAT: *r*(298) = 0.21, *p* < 0.001 *BAT: *r*(298) = −0.61, *p* < 0.001	Sequence‐based memory vs. beat alignment
Production	Dim 1	64.55%	PM1: *r*(296) = 0.91, *p* < 0.001 PM2: *r*(296) = 0.88, *p* < 0.001 UnP: *r*(296) = 0.58, *p* < 0.001	General tapping precision
	Dim 2	27.18%	UnP: *r*(296) = 0.82, *p* < 0.001 *PM2: *r*(296) = −0.34, *p* < 0.001 *PM1: *r*(296) = −0.19, *p* < 0.001	Paced vs. unpaced tapping
	Dim 3	8.28%	PM2, *r*(296) = 0.34, *p* < 0.001 *PM1, *r*(296) = −0.36, *p* < 0.001	Complex beat extraction

*Note*: * indicates negative contributions. Tests are presented in order of greatest contribution (positive contributions presented first). Degrees of freedom are calculated as *n* − 2 (*n* = 300 for perception PCA and 298 for production PCA).

Abbreviations: BAT = beat alignment test; BBA = beat‐based advantage task; BbMAT = Burgundy best Musical Aptitude Test synchronization task; Dim = Dimension; PM1 = paced tapping to Music 1; PM2 = paced tapping to Music 2; UnP = Unpaced Tapping.

### 3.3 Hierarchical Cluster Analysis—Perception Tasks

The hierarchical cluster analysis (HCA; see Figure 2A and Table 2) revealed three participant clusters. Performance on all tasks (all *p* values < 0.001) and all dimensions (all *p* values < 0.001) significantly contributed to the clusters. Cluster 1 (*n* = 112) performed inaccurately across all perception tasks, and were inaccurate along Dimension 1, relating to general rhythm performance, and Dimension 2, which positively loaded onto BbMAT performance. Therefore, Cluster 1 can be labeled as *weak perceivers*. Note that by weak perceivers, we refer to weak perceivers of the beat generally. For clarity with the other labels, we will refer to them as weak perceivers.

**TABLE 2:** All clusters, contributing tasks, and contributing dimensions for the perception PCA and the production PCA.

PCA	Clusters	Contributing tasks	Contributing dimensions
Perception	C1 (*n* = 112) *Weak perceivers*	*BbMAT: *v* = −11.61, *p* < 0.001 *BAT: *v* = −8.81, *p* < 0.001 *BBA: *v* = −6.56, *p* < 0.001	*Dim 1: *v* = −12.53, *p* < 0.001 *Dim 2: *v* = −5.31, *p* < 0.001
	C2 (*n* = 109) *Strong beat‐based perceivers*, *weak memory‐based perceivers*	BbMAT: *v* = 8.96, *p* < 0.001 BAT: *v* = 3.53, *p* < 0.001 *BBA: *v* = −6.02, *p* < 0.001	Dim 2: *v* = 10.89, *p* < 0.001 Dim 1: *v* = 2.54, *p* = 0.01 *Dim 3: *v* = −4.87, *p* < 0.001
	C3 (*n* = 79) *Strong perceivers, esp. sequence‐based memory and BAT*	BBA: *v* = 13.78, *p* < 0.001 BAT: *v* = 5.82, *p* < 0.001 BbMAT: *v* = 2.97, *p* = 0.003	Dim 1: *v* = 10.99, *p* < 0.001 Dim 3: *v* = 5.92, *p* < 0.001 *Dim 2: *v* = −6.06, *p* < 0.001
Production	C1 (*n* = 8) *Weak Tappers*	*UnP: *v* = −13.81, *p* < 0.001 *PM1: *v* = −5.23, *p* < 0.001 *PM2: *v* = −3.13, *p* = 0.002	*Dim 2: *v* = −11.31, *p* < 0.001 *Dim 1: *v* = −7.99, *p* < 0.001
	C2 (*n* = 101) *Weak beat‐based tappers*	*PM2: *v* = −13.29, *p* < 0.001 *PM1: *v* = −11.71, *p* < 0.001	Dim 2: *v* = 7.08, *p* < 0.001 *Dim 1: *v* = −11.89, *p* < 0.001
	C3 (*n* = 189) *Strong tappers*	PM2: *v* = 14.11, *p* < 0.001 PM1: *v* = 13.26, *p* < 0.001 UnP: *v* = 5.76, *p* < 0.001	Dim 1: *v* = 14.37, *p* < 0.001 *Dim 2: *v* = −3.16, *p* = 0.002

*Note*: * indicates to negative contributions. Tests are presented in order of greatest contribution (positive contributions presented first).

Abbreviations: BAT = beat alignment test; BBA = beat‐based advantage task; BbMAT = Burgundy best Musical Aptitude Test synchronization task; C = cluster; Dim = dimension; esp. = especially; PM1 = paced tapping to Music 1; PM2 = paced tapping to Music 2; UnP = unpaced tapping.

Cluster 2 (*n* = 109) performed well on the BbMAT and the BAT, and inaccurately on the BBA. Relatedly, this cluster performed well along Dimensions 2 and 1, but inaccurately across Dimension 3, which appears related to sequence‐based memory. Therefore, this cluster can be labeled as *strong beat‐based perceivers*, *weak memory‐based perceivers*.

Cluster 3 (*n* = 79) performed well across all perception tasks, and Dimensions 1 and 3, suggesting strong overall rhythm perception as well as strong sequence‐based memory perception. However, they had weaker performance on Dimension 2, suggesting higher BBA performance and lower BbMAT performance. This cluster could be labeled as *strong perceivers*, with the caveat that they might be stronger for sequence‐based memory and BAT compared to BbMAT, with the BbMAT containing more complex material.

**FIGURE 3:**
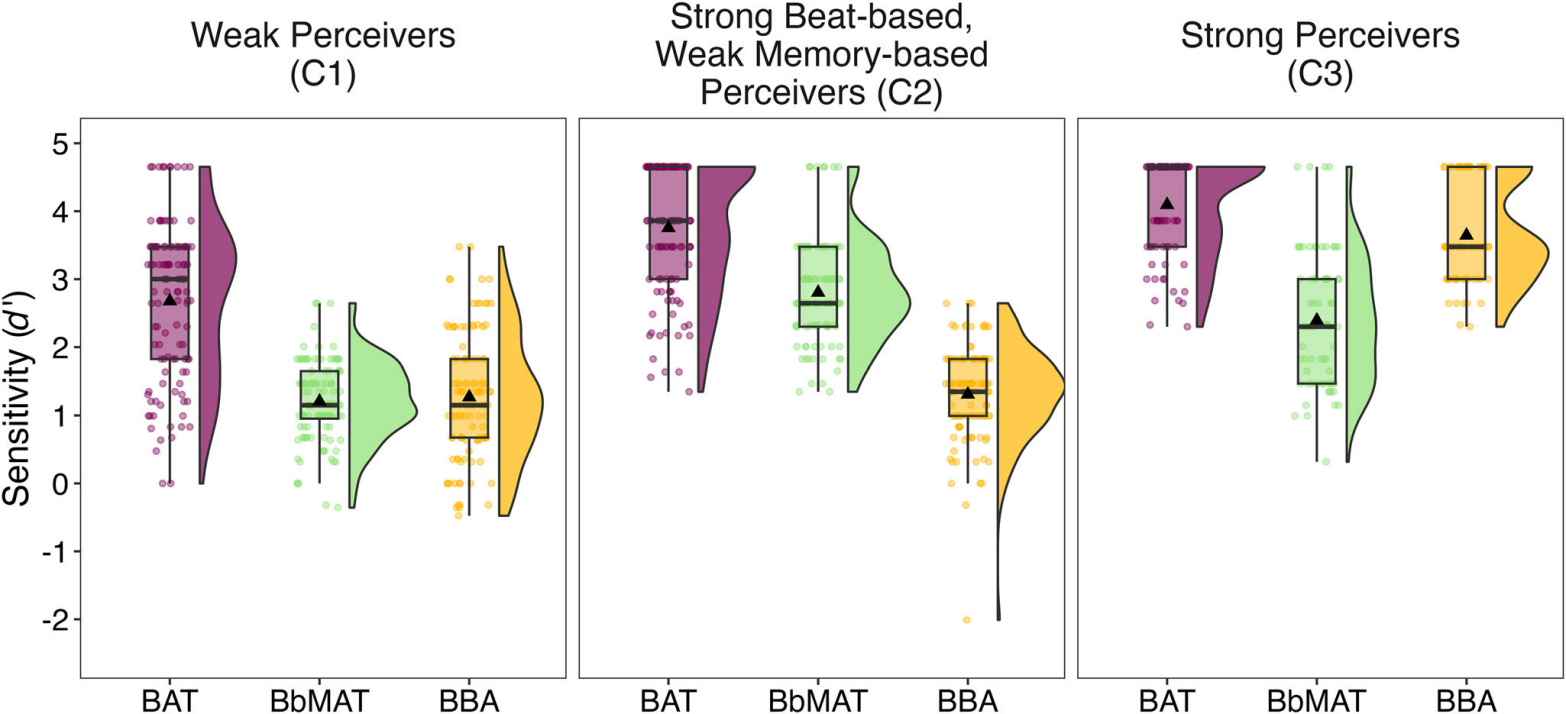
Performance on each perception task depending on the clusters (C1, C2, C3) that emerged from the perception‐only principal component analysis. See Table 3 for statistics. Boxplots show the median as the horizontal black line, the mean as the black triangle, and the interquartile range as the box. The violin plot shows the density of responses, and each dot is an individual data point. BAT = beat alignment test; BBA = beat‐based advantage task; BbMAT = Burgundy best Musical Aptitude Test synchronization task.

ANOVAs confirmed significant main effects of cluster on each task (see Figure 3): BBA, *F*(2, 188.39) = 290.44, *p* < 0.001, ω^2^ = 0.75, 95% CI [0.70, 0.79], BAT, *F*(2, 197.01) = 53.82, *p* < 0.001, ω^2^ = 0.35, 95% CI [0.24, 0.44], BbMAT, *F*(2, 170.75) = 171.09, *p* < 0.001, ω^2^ = 0.66, 95% CI [0.58, 0.72]. See Table 3 for all comparison statistics. The *strong perceivers* (Cluster 3) performed significantly better than the *weak perceivers* (Cluster 1) on all perception tasks. The *strong perceivers* also performed significantly better than the *strong beat‐based perceivers, weak memory‐based perceivers* (Cluster 2) for the BBA (*d* = 3.28), and for the BAT, but with a smaller effect size (*d* = 0.40). For the BbMAT, the reverse pattern was shown, with the *strong beat‐based, weak memory‐based perceivers* outperforming the *strong perceivers*, also with a smaller effect size. The *strong beat‐based, weak memory‐based perceivers* performed better than the *weak perceivers* (Cluster 1) on the BAT and the BbMAT, but not on the BBA.

**TABLE 3:** Cluster comparisons for the perception (Perc) and production (Prod) principal component analyses for all tests.

PCA	Cluster comparison		
Perc	*S perceivers* > *W perceivers*	BAT	Est = 1.42, *p*′ < 0.001, *d* = 1.45
	*S perceivers* > *W perceivers*	BbMAT	Est = 1.19, *p*′ < 0.001, *d* = 1.52
	*S perceivers* > *W perceivers*	BBA	Est = 2.37, *p*′ < 0.001, *d* = 2.88
	*S perceivers > S beat‐based, W memory‐based perceivers*	BAT	Est = 0.34, *p*′ = 0.02, *d* = 0.40
	*S perceivers < S beat‐based, W memory‐based perceivers*	BbMAT	Est = 0.41, *p*′ = 0.005, *d* = 0.48
	*S perceivers > S beat‐based, W memory‐based perceivers*	BBA	Est = 2.33, *p*′ < 0.001, *d* = 3.28
	*S beat‐based, W memory‐based perceivers > W perceivers*	BAT	Est = 1.08, *p*′ < 0.001, *d* = 1.01
	*S beat‐based, W memory‐based perceivers > W perceivers*	BbMAT	Est = 1.60, *p*′ < 0.001, *d* = 2.40
	*S beat‐based, W memory‐based perceivers = W perceivers*	BBA	Est = 0.04, *p*′ = 0.93, *d* = 0.05
Prod	*S tappers > W tappers*	Unpaced	Est = 0.31, *p*′ < 0.001, *d* = 3.75
	*S tappers > W tappers*	PM1	Est = 0.29, *p*′ = 0.001, *d* = 2.89
	*S tappers > W tappers*	PM2	Est = 0.27, *p*′ = 0.001, *d* = 2.60
	*S tappers > W beat‐based tappers*	Unpaced	Est = 0.02, *p*′ < 0.001, *d* = 0.67
	*S tappers > W beat‐based tappers*	PM1	Est = 0.19, *p*′ < 0.001, *d* = 2.19
	*S tappers > W beat‐based tappers*	PM2	Est = 0.27, *p*′ < 0.001, *d* = 2.79
	*W beat‐based tappers > Weak Tappers*	Unpaced	Est = 0.29, *p*′ < 0.001, *d* = 3.46
	*W beat‐based tappers = Weak Tappers*	PM1	Est = 0.11, *p*′ = 0.15, *d* = 0.84
	*W beat‐based tappers = Weak Tappers*	PM2	Est = 0.002, *p*′ = 0.998, *d* = 0.02

*Note*: See Figure 3.

Abbreviations: BAT = beat alignment test; BBA = beat‐based advantage task; BbMAT = Burgundy best Musical Aptitude Test synchronization task; Est = estimate; PM1 = paced tapping to Music 1; PM2 = paced tapping to Music 2; S = strong; W = weak.

Years of musical training significantly differed depending on cluster, *F*(2, 156.09) = 5.91, *p* = 0.003, ω^2^ = 0.06, 95% CI [0.00, 0.14], which appeared primarily driven by a significant difference in musical training between the *weak perceivers* (Cluster 1; *M* = 0.66 years, *SD* = 1.75 years, range = 0–10 years) who had less musical training than the s*trong perceivers* (Cluster 3; *M* = 2.22 years, *SD* = 3.81 years, range = 0–15 years), Est = 1.55, *p* = 0.003, *d* = 0.53. The difference between the *strong perceivers* (Cluster 3) and the *strong beat‐based, weak memory‐based perceivers* (Cluster 2; *M* = 1.08 years, *SD* = 3.26 years, range = 0–25 years) did not reach significance, Est = 1.13, *p* = 0.086, *d* = 0.32, and there was no difference in musical training between the *weak perceivers* and the *strong beat‐based, weak memory‐based perceivers* (Clusters 1 and 2), Est = 0.42, *p* = 0.46, *d* = 0.16.

The multinomial logistic regression achieved very high classification accuracy using the leave‐one‐out method. The model correctly identified 99.33% of participants into their respective clusters (κ = 0.99, 95% CI [0.98, 0.999]), significantly above the 37.33% expected by chance (*p* < 0.001). Only two participants were misclassified (0.67%): one from Cluster 1 misclassified as Cluster 3, and one from Cluster 3 misclassified as Cluster 2.

### 3.4 Principal Component Analysis—Production Tasks

Three components emerged from the production PCA, accounting for 100% of the variance. See Table 1 for statistical details and Figure 2B for visualizations. Dimension 1 positively correlated with performance on all tapping tasks, reflecting reduced tapping variability. Dimension 2 positively correlated with unpaced tapping, and negatively with Paced Music 2 (PM2) and Paced Music 1 (PM1). This dimension appears to reflect a distinction between precision when internally generating a beat compared to synchronizing with an external beat. Dimension 3 positively correlated with PM2, and negatively with PM1, suggesting a distinction between the two types of music, with PM1 resulting in higher performance than PM2.

### 3.5 Hierarchical Cluster Analysis—Production

The HCA revealed three participant clusters (see Figure 2B and Table 2). All tasks significantly contributed to the clusters (all *p* values < 0.001), as did Dimension 1 and Dimension 2 (*p* values < 0.001). Cluster 1 (*n* = 8) was a small cluster containing participants with high tapping variability (based on their CV of ITIs) across all tapping tasks. This cluster negatively correlated with performance on Dimension 2, which was driven by variable performance on the unpaced tapping task, as well as Dimension 1, considered as general tapping precision. Cluster 1 could be labeled as particularly *weak tappers*.

Cluster 2 (*n* = 101) had high variability specifically for PM2 and PM1, suggesting a particular difficulty in synchronizing with an external beat that needs to be extracted from musical structure. This result is in line with a positive contribution of Dimension 2, which negatively loaded onto the paced tapping tasks and positively loaded on the unpaced tapping tasks. Further, Dimension 1 (general tapping precision) negatively correlated with Cluster 2, further suggesting poor tapping performance. This cluster could be considered as *weak beat‐based tappers*.

Cluster 3 (*n* = 189) had low variability across all tapping tasks, positively correlating with Dimension 1, and negatively with Dimension 2. Globally, this cluster appears to be *strong tappers*, with a slight benefit for the paced tapping tasks compared to unpaced tapping.

**FIGURE 4:**
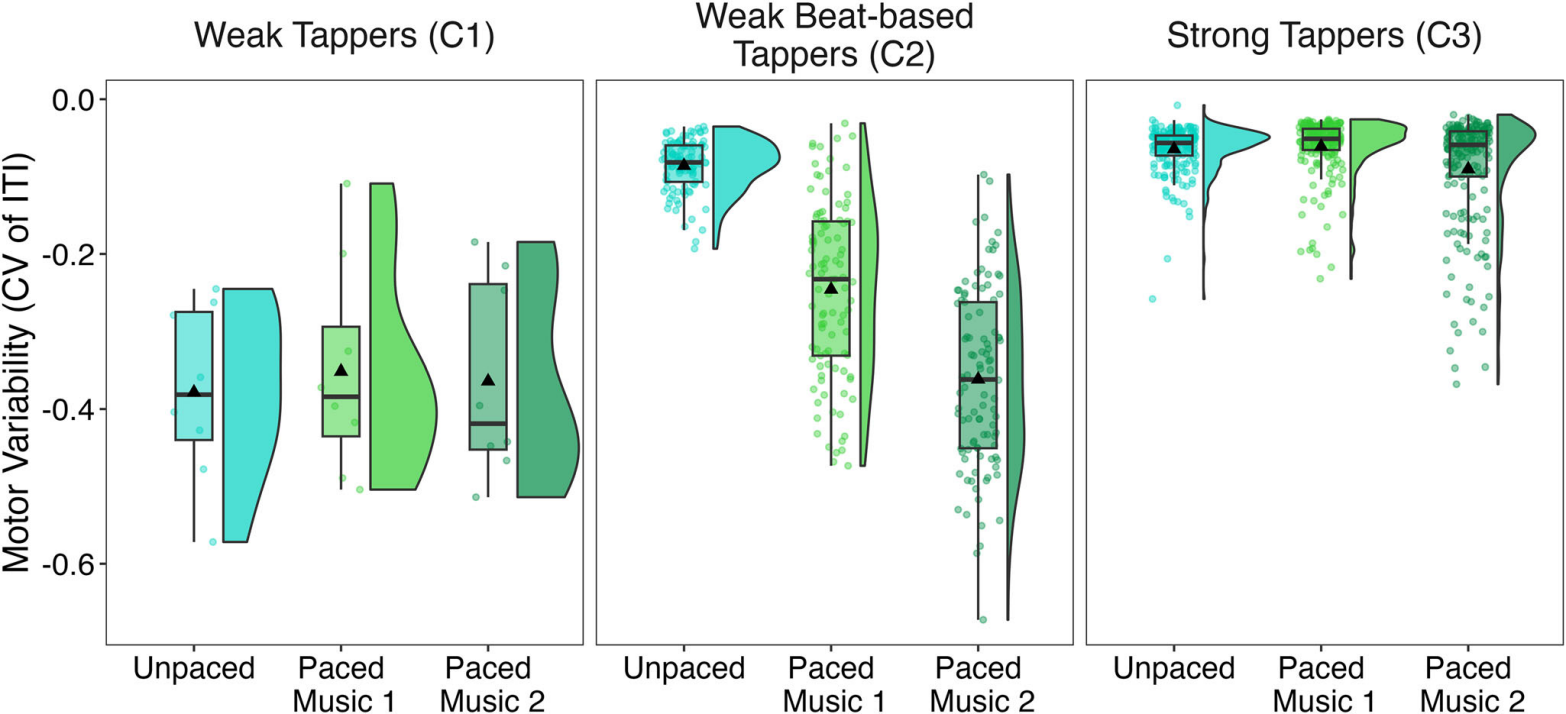
Performance on each production task depending on the clusters (C1, C2, C3) that emerged from the production‐only principal component analysis. Coefficient of variation of the intertap interval (CV of ITI, reflecting motor variability) values have been reversed, so that more positive values reflect less variability in tapping. Boxplots show the median as the horizontal black line, the mean as the black triangle, and the interquartile range as the box. The violin plot shows the density of responses, and each dot is an individual data point.

ANOVAs confirmed main effects of cluster for unpaced tapping (see Figure 4), *F*(2, 18.05) = 41.44, *p* < 0.001, ω^2^ = 0.79, 95% CI [0.57, 0.88], PM1, *F*(2, 17.67) = 138.52, *p* < 0.001, ω^2^ = 0.93, 95% CI [0.85, 0.96], and PM2, *F*(2, 18.22) = 231.77, *p* < 0.001, ω^2^ = 0.96, 95% CI [0.90, 0.97]. The *strong tappers* (Cluster 3) were less variable in tapping (CVs of ITIs) than the *weak tappers* (Cluster 1) across all tapping tasks. See Table 3 for all comparison statistics. The *strong tappers* also performed better than the *weak beat‐based tappers* (Cluster 2) across all tapping tasks. The only difference between the *weak tappers* and the *weak beat‐based tappers* was for the unpaced tapping task, with the *weak beat‐based tappers* outperforming the *weak tappers*. There was no significant difference between the *weak tappers* and the *weak beat‐based tappers* for PM1 or PM2. However, it should be noted that the effect size was large for the difference in PM1 (*d* = 0.84). The lack of significance may be related to the unequal group sizes, with only eight participants in Cluster 1.

Years of musical training significantly differed depending on cluster, *F*(2, 19.90) = 4.97, *p* = 0.018, ω^2^ = 0.26, 95% CI [0.00, 0.51]. However, the only significant difference between groups was that the *strong tappers* (Cluster 3; *M* = 1.59 years, *SD* = 3.49 years, range = 0–25 years) had more years of training than the w*eak beat‐based tappers* (Cluster 2; *M* = 0.58 years, *SD* = 1.84 years, range = 0–10 years), Est = 1.00, *p* = 0.004, *d* = 0.36. There was no significant difference between the *strong tappers* (Cluster 3) and the *weak tappers* (Cluster 1*; M* = 1.00 years, *SD* = 1.93 years, range = 0–5 years), Est = 0.59, *p* = 0.71, *d* = 0.21 or between the *weak tappers* and the *weak beat‐based tappers*, Est = 0.42, *p* = 0.83, *d* = 0.22, likely due to the small sample size for the *weak tappers*.

The multinomial logistic regression achieved very high classification accuracy using the leave‐one‐out method. The model correctly identified 100% of participants into their respective clusters (κ = 1.00, 95% CI [0.99, 1.00]), significantly above the 63.42% expected by chance (*p* < 0.001). No participants were misclassified.

### 3.6 Principal Components Analysis—Combined Perception + Production

Six dimensions emerged from the combined perception and production PCA, accounting for 100% of the variance (see Figure 2C and Table 4). The first five dimensions accounted for 95.88% of the variance, and the sixth dimension accounted for less than 5% of the variance, so only five will be described. The sixth component was so small that loadings were not made available through *FactoMineR*. Dimensions 4 and 5 are reported in Supporting Information Section 2.1, Figure S5, and Table S3.

**TABLE 4:** Overview of the dimensions, variance explained, contributing factors, and interpretation of the principal components analyses (PCA) for the first three dimensions of the combined perception + production PCA.

PCA	Dim	Variance	Contributing tasks	Interpretation
Perception + production	Dim 1	40.02%	PM1: *r*(296) = 0.84, *p* < 0.001 PM2: *r*(296) = 0.80, *p* < 0.001 BAT: *r*(296) = 0.68, *p* < 0.001 UnP: *r*(296) = 0.52, *p* < 0.001 BBA: *r*(296) = 0.43, *p* < 0.001 BbMAT: *r*(296) = 0.38, *p* < 0.001	General rhythm performance (perception and production)
	Dim 2	17.54%	BBA: *r*(296) = 0.65, *p* < 0.001 BbMAT: *r*(296) = 0.49, *p* < 0.001 BAT: *r*(296) = 0.32, *p* < 0.001 *PM2: *r*(296) = −0.38, *p* < 0.001 *PM1: *r*(296) = −0.37, *p* < 0.001 *UnP: *r*(296) = −0.12, *p* = 0.03	Perception vs. production
	Dim 3	14.73%	BbMAT: *r*(296) = 0.73, *p* < 0.001 UnP: *r*(296) = 0.28, *p* < 0.001 *BBA: *r*(296) = −0.47, *p* < 0.001 *BAT: *r*(296) = −0.20, *p* < 0.001	Internal beat generation vs. external beat following

*Note*: * indicates negative contributions. Tests are presented in order of greatest contribution (positive contributions presented first). Degrees of freedom are calculated as *n* − 2 (*n* = 298).

Abbreviations: BAT = beat alignment test; BBA = beat‐based advantage task; BbMAT = Burgundy best Musical Aptitude Test synchronization task; Dim = dimension; PM1 = paced tapping to Music 1; PM2 = paced tapping to Music 2; UnP = unpaced tapping.

Dimension 1 positively correlated with performance on all perception and production tasks, and could be considered to reflect general rhythm performance (perception and production). Dimension 2 positively correlated with all perception variables, and negatively correlated with all production variables, reflecting a clear distinction between performance on perception versus production tasks. Dimension 3 positively correlated with BbMAT and unpaced tapping, and negatively correlated with BBA and BAT. It is possible that this dimension relates to internal beat generation compared to external beat following, considering that internal beat generation is necessary for unpaced tapping, and could be valuable in judging synchronization in BbMAT.

### 3.7 Hierarchical Cluster Analysis—Combined Perception + Production

The HCA revealed three participant clusters (see Figure 2C and Table 5). Performance on all tasks significantly contributed to the clusters (all *p* values < 0.001). Dimensions 1 (η^2^ = 0.75, *p* < 0.001), 2 (η^2^ = 0.44, *p* < 0.001), and 5 (η^2^ = 0.02, *p* = 0.03) significantly contributed to the clusters. Cluster 1 (*n* = 59) performed inaccurately across the perception measures of BAT and BBA, as well as all tapping measures. This cluster also performed inaccurately along Dimension 1 (and Dimension 5, see Supporting Information Section 2). However, they performed strongly along Dimension 2 which was the distinction between perception (positively loading) and production (negatively loading). This group could be considered to have *weak rhythm processing* (perception and production), and specifically weak production.

**TABLE 5:** All clusters, contributing tasks, and contributing dimensions for the combined perception + production hierarchical cluster analysis.

Perception + production	C1 (*n* = 59) *Weak rhythm processing (perception and production), specifically weak production*	*BAT: *v* = −4.48, *p* < 0.001 *BBA: *v* = −1.99, *p* = 0.047 *PM1: *v* = −14.25, *p* < 0.001 *PM2: *v* = −12.27, *p* < 0.001 *UnP: *v* = −6.63, *p* < 0.001	*Dim 1: *v* = −12.20, *p* < 0.001 *Dim 5: *v* = −2.58, *p* = 0.01 Dim 2: *v* = 7.41, *p* < 0.001
	C2 (*n* = 111) *Weak perceivers, good simple tappers*.	*BBA: *v* = −7.61, *p* < 0.001 *BAT: *v* = −7.13, *p* < 0.001 *BbMAT: *v* = −6.45, *p* < 0.001 PM1: *v* = 2.28, *p* = 0.02	*Dim 2: *v* = −10.90, *p* < 0.001 *Dim 1: *v* = −3.27, *p* = 0.001
	C3 (*n* = 128) *Strong rhythm perception/production*	BAT: *v* = 10.57, *p* < 0.001 PM2: *v* = 9.33, *p* < 0.001 PM1: *v* = 9.24, *p* < 0.001 BBA: *v* = 9.03, *p* < 0.001 BbMAT: *v* = 6.73, *p* < 0.001 UnP: *v* = 4.70, *p* < 0.001	Dim 1: *v* = 13.02, *p* < 0.001 Dim 2: *v* = 4.68, *p* < 0.001

*Note*: * indicates negative contributions. Tests are presented in order of greatest contribution (positive contributions presented first).

Abbreviations: BAT = beat alignment test; BBA = beat‐based advantage task; BbMAT = Burgundy best Musical Aptitude Test synchronization task; Dim = dimension; PM1 = paced tapping to Music 1; PM2 = paced tapping to Music 2; UnP = unpaced tapping.

Cluster 2 (*n* = 111) showed inaccurate performance on all perception measures but showed good performance on PM1. Related to their weak perception results, they performed inaccurately across Dimensions 2 and 1. This group could be considered as *weak perceivers, good simple tappers*.

Cluster 3 (*n* = 128) showed strong performance across all perception and production tasks. This cluster also positively related to Dimensions 1 and 2, suggesting strong rhythm skills. This group can be labeled as *strong rhythm perception/production*.

**FIGURE 5:**
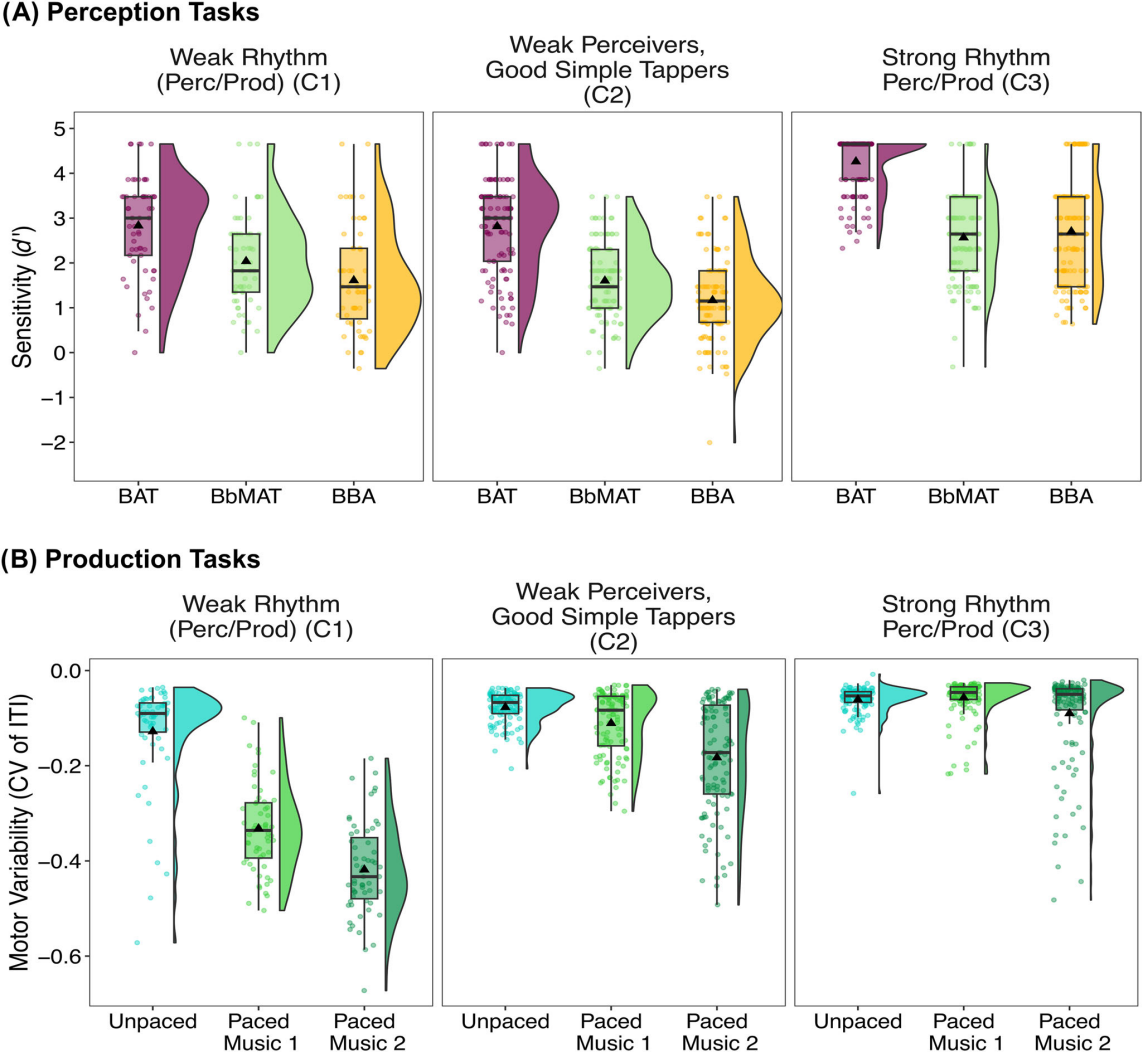
Performance on (A) perception and (B) production tasks depending on the clusters (C1, C2, C3) that emerged from the perception + production principal component analysis. See Table 6 for statistics. Coefficient of variation of the intertap interval (CV of ITI, reflecting motor variability) values have been reversed, so that more positive values reflect less variability in tapping. Boxplots show the median as the horizontal black line, the mean as the black triangle, and the interquartile range as the box. The violin plot shows the density of responses, and each dot is an individual data point. BAT = beat alignment test; BBA = beat‐based advantage task; BbMAT = Burgundy best Musical Aptitude Test synchronization task.

ANOVAs confirmed significant effects of clusters across all tasks (see Figure 5), both for perception: BBA, *F*(2, 150.75) = 61.42, *p* < 0.001, ω^2^ = 0.44, 95% CI [0.32, 0.53], BAT: *F*(2, 131.53) = 101.76, *p* < 0.001, ω^2^ = 0.60, 95% CI [0.50, 0.68], BbMAT: *F*(2, 295) = 31.40, *p* < 0.001, ω^2^ = 0.17, 95% CI [0.10, 0.24], and production: unpaced tapping: *F*(2, 124.65) = 16.99, *p* < 0.001, ω^2^ = 0.20, 95% CI [0.08, 0.32], PM1, *F*(2, 124.1) = 248.06, *p* < 0.001, ω^2^ = 0.80, 95% CI [0.74, 0.84], and PM2: *F*(2, 155.37) = 229.34, *p* < 0.001, ω^2^ = 0.74, 95% CI [0.68, 0.79]. See Table 6 for all comparison statistics.

**TABLE 6:** Cluster comparisons for the perception + production (Perc + Prod) principal component analysis for all tests.

PCA	Cluster comparison		
Perc	*S perc/prod >* *W perc, good simple tappers*	BAT	Est = 1.45, *p*′ < 0.001, *d* = 1.62
+	*S perc/prod >* *W perc, good simple tappers*	BbMAT	*t*(237) = 8.19, *p*′ < 0.001, *d* = 1.06
Prod	*S perc/prod >* *W perc, good simple tappers*	BBA	Est = 1.54, *p*′ < 0.001, *d* = 1.42
	*S perc/prod >* *W perc, good simple tappers*	Unpaced	Est = 0.02, *p*′ < 0.001, *d* = 0.54
	*S perc/prod >* *W perc, good simple tappers*	PM1	Est = 0.05, *p*′ < 0.001, *d* = 0.97
	*S perc/prod >* *W perc, good simple tappers*	PM2	Est = 0.09, *p*′ < 0.001, *d* = 0.87
	*S perc/prod > W rhythm*	BAT	Est = 1.43, *p*′ < 0.001, *d* = 1.61
	*S perc/prod > W rhythm*	BbMAT	*t*(185) = 3.35, *p*′ = 0.002, *d* = 0.53
	*S perc/prod > W rhythm*	BBA	Est = 1.09, *p*′ < 0.001, *d* = 0.92
	*S perc/prod > W rhythm*	Unpaced	Est = 0.07, *p*′ < 0.001, *d* = 0.82
	*S perc/prod > W rhythm*	PM1	Est = 0.28, *p*′ < 0.001, *d* = 3.86
	*S perc/prod > W rhythm*	PM2	Est = 0.33, *p*′ < 0.001, *d* = 3.39
	*W perc, good simple tappers = W rhythm*	BAT	Est = 0.02, *p*′ = 0.995, *d* = 0.01
	*W perc, good simple tappers < W rhythm*	BbMAT	*t*(168) = 2.95, *p*′ = 0.004, *d* = 0.48
	*W perc, good simple tappers < W rhythm*	BBA	Est = 0.44, *p*′ = 0.03, *d* = 0.42
	*W perc, good simple tappers > W rhythm*	Unpaced	Est = 0.05, *p*′ = 0.003, *d* = 0.61
	*W perc, good simple tappers > W rhythm*	PM1	Est = 0.22, *p*′ < 0.001, *d* = 2.72
	*W perc, good simple tappers > W rhythm*	PM2	Est = 0.24, *p*′ < 0.001, *d* = 2.18

*Note*: See Figure 5.

Abbreviations: BAT = beat alignment test; BBA = beat‐based advantage task; BbMAT = Burgundy best Musical Aptitude Test synchronization task; Est = estimate; PM1 = paced tapping to Music 1; PM2 = paced tapping to Music 2; S = strong; W = weak.

Across all tests, the *strong rhythm perception/production* cluster (Cluster 3) performed better than the *weak perceivers, good simple tappers* (Cluster 2) not only for the perception tasks, but also for the tapping tasks, though with smaller effect sizes. The *strong rhythm perception/production* cluster also outperformed the group with *weak rhythm processing* (Cluster 1) on all perception tasks and all production tasks.

There were different patterns of results between Cluster 2 (*weak perceivers, good simple tappers*) and Cluster 1 (*weak rhythm processing*). For the perception tasks, the *weak rhythm* group performed better than the *weak perceivers, good simple tappers* for BBA and BbMAT, but not for the BAT. However, for the production tasks, the reverse pattern was found: the *weak perceivers, good simple tappers* performed better than the *weak rhythm* group for all tapping tasks. These results show that for the perception tasks, the *weak rhythm processing* participants were outperforming those with specifically weak perception, whereas for the production tasks, the *weak perceivers, good simple tappers* were outperforming the *weak rhythm* participants.

Years of musical training significantly differed depending on cluster, *F*(2, 175.77) = 4.97, *p* = 0.008, ω^2^ = 0.04, 95% CI [0.00, 0.11], which appeared to be driven by the *strong rhythm perception/production* cluster (Cluster 3*; M* = 1.91 years, *SD* = 3.99 years, range = 0–25 years) who had more musical training than both the *weak rhythm* cluster (Cluster 1; *M* = 0.64 years, *SD* = 1.71 years, range = 0–10 years), Est = 1.26, *p* = 0.008, *d* = 0.41, and the *weak perceivers, good simple tappers* (Cluster 2*; M* = 0.77 years, *SD* = 1.99 years, range = 0–10 years), Est = 1.14, *p* = 0.013, *d* = 0.36. There was no difference between the *weak rhythm* cluster and the *weak perceivers, good simple tappers* group, Est = 0.12, *p* = 0.91, *d* = 0.07.

The multinomial logistic regression achieved very high classification accuracy using the leave‐one‐out method. The model correctly identified 98.32% of participants into their respective clusters (κ = 0.97, 95% CI [0.96, 0.99]), significantly above the 42.95% expected by chance (*p* < 0.001). Only five participants were misclassified (1.7%), with the most classification errors between Clusters 1 and 2 (three participants).

### 3.8 Links With Questionnaire Data

A multinomial logistic regression including age, education (ordered four levels), and years of music training as factors investigated whether the responses to the four music statements: (1) “I can tap in time with a musical beat”; (2) “I am a good dancer; I dance in rhythm with the music”; (3) “I have trouble recognizing songs without the lyrics”; (4) “I am rarely able to notice if someone is singing out of tune” could predict allocation to Cluster 1 (*weak rhythm processing, n* = 59), Cluster 2 (*weak perceivers, good simple tappers, n =* 111) or Cluster 3 (*strong rhythm perception/production, n* = 128) from the combined perception and production PCA. Responses to negatively scored statements were reversed so that higher scores always related to better performance. The average music score significantly predicted cluster grouping, χ ^2^(2, *N* = 298) = 15.43, *p* < 0.001, as did years of music training, χ ^2^(2) = 6.50, *p* = 0.04. Age failed to reach significance, χ ^2^(2) = 5.45, *p* = 0.065, and there was no effect of education (*p* = 0.93). These results suggest a relationship between subjective responses to music statements and objective task performance. To investigate further, exploratory correlational analyses were run on the self‐evaluation of music skills and task performance across the six tasks. All results are shown in Supporting Information Section 4 and Figure S7. After controlling for multiple correlations, responses to the statement: “I can tap in time with a musical beat” positively correlated with performance on the BAT, BBA, PM1, and PM2 (supporting Niarchou et al. [58]), and responses to the statement “I am rarely able to notice if someone is singing out of tune” negatively correlated with performance on the BAT, BBA, and PM1, suggesting that participants with higher scores on this amusic trait statement had lower scores on the rhythm tasks (see Table S5 for all information; in agreement with van Vugt et al. [69]). Such results support research showing correlations between objective task performance and self‐report measures [60].

## 4 Discussion

The current study aimed to replicate and extend the results of the lab‐based study in Fiveash et al. [3], to investigate whether distinct rhythmic competencies would also emerge in a larger, internet‐based sample of participants completing different rhythm perception and production tasks. Similar distinctions between perception and production tasks, as well as between beat‐based and sequence memory‐based rhythm perception tasks emerged as in Fiveash et al. [3]. However, the result patterns were different in this larger sample, and an additional distinction between unpaced and paced tapping tasks emerged, supporting previous findings in the literature (e.g., Ref. [4]). In contrast to Fiveash et al. [3], who showed distinctions already in the first dimensions of the PCAs, the current results showed that the first and largest dimension in each PCA always accounted for variance across all tasks. This pattern was also reflected in the HCAs, with one cluster in each PCA reflecting participants who performed well across all tasks and one cluster reflecting participants who performed weakly across all tasks. However, interesting clusters of participants emerged who were selectively strong or weak at different tasks, suggesting different patterns of rhythmic competencies across tasks.

The current results suggest that with larger samples, different types of rhythm tasks do appear to tap into similar underlying processing skills; however, distinct clusters of participants also emerged with different rhythmic profiles. These individual differences are likely to have a larger influence on results in smaller samples and might be particularly influential and informative when investigating clinical populations with potential impairments in rhythm perception and/or production skills.

Going beyond the initial dimensions, and at the individual participant level, interesting distinctions emerged. In the combined PCA, a clear distinction emerged between the perception and production tasks, which was also reflected with a cluster of participants (*n* = 111) who performed poorly on the three perception tasks, but well on the simple paced tapping to music task (PM1). This pattern aligns with reports of participants who showed poor rhythm perception, but typical rhythm production [2]. It was interesting that this cluster did not show correlations with the PM2 task, or the unpaced tapping task, suggesting that in more complex beat extraction situations (PM2) or when self‐generating a beat, they did not show particular strengths or weaknesses. However, they still performed better than the *weak rhythm* group on all tapping tasks. In the perception‐only PCA, a cluster (*n* = 109) emerged who had strong beat‐based perception, but weak memory‐based perception, suggesting a distinction related specifically to weak memory for rhythmic sequences. This result supports previous work showing distinctions between beat‐based and memory‐based tasks [3, 5, 6, 7, 14], though it is surprising that the distinction emerged here only at the cluster level and not within the PCAs, as in Fiveash et al. [3].

The production‐only PCA showed a clear distinction between the two paced tapping tasks and the unpaced tapping task, which was also reflected in a cluster of participants (*n* = 101) who were particularly weak at the paced tapping tasks, but not the unpaced tapping task. This pattern was also found in Sowínski and Dalla Bella [4] (*n* = 99), who found that poor synchronizers performed normally for unpaced (spontaneous) tapping. They interpreted this distinction as a potential alteration of auditory‐motor coupling, as opposed to motor implementation. This distinction between unpaced and paced tapping tasks could also be relevant to clinical populations. For example, children with developmental language disorder showed impaired performance compared to a control group on paced but not unpaced tapping [70], though it should be noted that performance was low overall, perhaps also reflecting some floor effects. Other studies have shown that children and adults with ADHD were impaired in both unpaced and paced tapping tasks, but that this difference was not related to motor variability [21]. Interestingly, a small cluster of participants emerged (*n* = 8) who performed poorly across all tapping tasks, showing a larger impairment in tapping variability across both unpaced and paced tapping tasks.

The finding that all rhythm tasks loaded onto the first and largest dimension of each PCA, even within the combined perception and production PCA, was somewhat surprising, and could have different interpretations. It is notable that this pattern of results was not observed in the smaller, lab‐based sample of Fiveash et al. [3], and could incorporate different sources of variance, including general rhythmic skills, as well as general cognitive skills. These possibilities cannot be distinguished in the current sample as nonrhythmic tasks were not included. Considering the distinctions across tests observed in the second and third dimensions, as well as the distinct participant clusters emerging with different result patterns, it is possible that both types of skills could be contributing to the results to a certain extent.

In either case, the results suggest that within a larger participant sample, different rhythm tasks are capturing some shared variance in performance that was not captured in the smaller participant sample [3]. If the first dimensions are largely reflecting general rhythm‐related skills, then with larger sample sizes, any of the rhythm tests used here would be likely to tap into these general underlying rhythmic competencies (as could be seen in larger‐scale genetic studies [28, 34, 58]). However, general, nonrhythm‐related mechanisms, such as executive functioning, WMC, or general intelligence, might influence performance too and be reflected here. Future research should include some measures of nonrhythmic capacity, such as WMC or executive functions to tease apart these possibilities. It would be particularly interesting to further investigate connections between WMC and the memory‐based rhythm task reported here.

We completed our analyses by exploring some potential links with the questionnaire data. The music‐related questionnaire responses predicted cluster grouping in the combined PCA, including years of musical training, suggesting that self‐report measures for music may be suitable proxies for measured task performance. Responses to the statement “I can tap in time with a musical beat” correlated with paced tapping to Music 1 and 2, as well as the BAT, and to a lesser extent, the BBA, supporting results in a larger sample showing a correlation with responses to this statement and objective rhythm task performance [58]. A supplementary analysis (Supporting Information Section 2.2) that included the production task performance and responses to the two statements, “I can tap in time with a musical beat” and “I'm a good dancer; I dance in rhythm with the music,” showed distinct clusters of participants who were (a) weak at production tasks, and knew it (*n* = 54); (b) strong at production tasks, and knew it (*n* = 107, also showing more musical training than the other groups); and (c) strong at production tasks, but did not know it (*n* = 137). This pattern suggests that although across large samples of participants whereby self‐report measures globally reflect objective performance, there could be a large group of participants where self‐report measures do not capture objective rhythmic skills. As might be expected, objective performance across tasks related to years of musical training. Cluster grouping significantly related to years of musical training across all principal components analyses (as seen in Dalla Bella et al. [13]). The strong‐beat perceivers (perception PCA), strong tappers (production PCA), and *strong rhythm perception/production* cluster (combined PCA) all had more years of music training than the other clusters.

### 4.1 Limitations and Future Directions

Due to time constraints for attention on the internet‐based testing platform, we chose to reduce the number of tests to those that emerged as the most distinctive in Fiveash et al. [3] and based on distinctions in the literature. However, this meant that there were only three perception tasks (BAT, BbMAT, BBA) and two production tasks (unpaced tapping, paced tapping to Music 1 and 2). Although we did not include a large battery of tests, recent work has shown that smaller subsets of tasks may capture much of the variance of larger test batteries. For example, Dalla Bella et al. [13] showed that variance across the BAASTA test, taking more than 2 h, could be largely captured using two perception tasks (beat alignment task with a 750 ms inter‐onset interval, and the anisochrony detection task) and two production tasks (paced tapping to music and paced tapping to metronome) tasks. Further, Dalla Bella et al. [11] proposed that a beat tracking index composite score made up of the BAT and paced tapping to music could largely reflect variance captured by the longer test battery. Together with our findings, these results suggest that including a measure of paced tapping and a measure of beat alignment, plus a memory‐based task, might be sufficient to capture most of the variation in rhythm competencies. This minimal test battery could then be tested across different clinical populations, for example, in a multilab set‐up such as in Grassi et al. [71], to be able to access a larger number of participants, or to focus on difficult‐to‐reach populations. Based on this future research, it might then be possible to test larger samples with the aim to create norms and thresholds for what constitutes a “typical” range of performance for different tasks.

It is also possible that our current selection of rhythm tasks did not cover all relevant influences on rhythm perception and production. For example, we did not measure WMC, which might have been interesting in line with the contribution to the memory‐based rhythm task (e.g., Drakoulaki et al. [6]). Further, we did not have a rhythm reproduction task, as we wanted to isolate rhythm memory specifically; however, previous research has shown distinctions between rhythm reproduction and tapping/clapping in time [5, 7]. Future research might consider links between performance across the different rhythm perception and production tasks, and how they might relate to performance on other cognitive tests and questionnaires, with particular focus on individual differences in language skills.

Another interesting question relates to the links between musical training and task performance. Our results showed that self‐reported years of musical training was associated with cluster membership. However, this self‐report measure might not capture different musical skills. Future research could consider using previously proposed measures of musical sophistication (such as the Goldsmiths Musical Sophistication Index) [60], or another short assessment of musical skills such as the micro profile of music perception skills (micro‐PROMS) [72, 73].

Finally, the finding that distinctions between tasks and between clusters emerged with internet‐based testing shows that online testing may be a promising method to investigate rhythmic competencies in the future (see also Refs. [52, 58]). For example, internet‐based testing would allow for data collection from very large samples, as well as difficult‐to‐reach populations, including clinical populations with small participant pools. Large‐scale approaches, such as using citizen science with gamified implementations [74] could be particularly valuable for this approach.

## 5 Conclusion

Taken together, the current results support findings from Fiveash et al. [3], showing evidence for separable rhythmic competencies across an internet‐based sample of 300 participants. Separations in terms of dimensions and clusters were found between perception and production tasks, and unpaced and paced tapping tasks. Further, a cluster of participants emerged who showed strong beat‐based processing, but weak sequence memory‐based processing. In this larger participant sample, the first dimension across each PCA accounted for variance across all tasks, suggesting a common, underlying rhythmic or general nonrhythmic ability across participants. The current findings also support the use of internet‐based testing for measuring different rhythmic competencies in larger populations. Such implementations could be particularly valuable for difficult‐to‐reach clinical populations, as well as providing the basis to develop and test such implementations with school‐age children, specifically relevant in link with language development and pathology. The current results suggest that it is valuable to include a production task, a beat‐based rhythm perception task, and a sequence memory‐based rhythm task when measuring rhythmic competencies in both typical and clinical populations.

## Author Contributions

Anna Fiveash: conceptualization, methodology, software, formal analysis, investigation, data curation, writing – original draft, writing – review and editing, visualization. Nicholas E. V. Foster: methodology, software, validation, formal analysis, investigation, resources, data curation, writing – review and editing. Reyna L. Gordon: conceptualization, methodology, resources, writing – review and editing. Simone Dalla Bella: conceptualization, methodology, resources, writing – review and editing. Barbara Tillmann: conceptualization, methodology, resources, writing – review and editing, supervision, project administration, funding acquisition.

## Conflicts of Interest

Simone Dalla Bella is on the board of the BeatHealth Company, which is dedicated to the design and commercialization of technological tools for assessing rhythm skills such as the BAASTA tablet and implementing rhythm‐based interventions. The other authors declare no conflicts of interest.

## Supporting information




**Supporting Information**: nyas70372‐sup‐0001‐SuppMat.docx

## Data Availability

All data and scripts used in the current analysis are available here: https://osf.io/4r5hd/overview?view_only=58f4733b79a84624928edd59c25d6ef0.
