# Supplementary material for: Distinct Rhythmic Competencies Identified via Internet‐Based Assessment
Source: Ann N Y Acad Sci. 2026 Aug 11;1562(1):e70372. doi: 10.1111/nyas.70372 (PMC13460242; doi:10.1111/nyas.70372)
Supplement: Supplementary file 1 [file NYAS-1562-0-s001.docx]

**Supplementary Material for:**

**Fiveash, Foster, Gordon, Dalla Bella & Tillmann (2026).**

***Distinct rhythmic competencies identified via internet-based assessment***

1. **Task Performance Measures**
   1.1. **Perception Tasks**

Table S1 outlines descriptive statistics for all perception tasks. Statistics for differences between conditions are reported below for *d’* and response bias *c*. Accuracy rates and hits minus false alarms are reported for completion.

| **Table S1**  *Accuracy, Hits-FA, D prime, and Response Bias c for the Perception Tasks* | | | | | | | | | |
| --- | --- | --- | --- | --- | --- | --- | --- | --- | --- |
|  |  | BBA | | | BAT | | BbMAT-S | | |
|  |  | Simple | Complex | All | Phase | Period | All |  |  |
| Accuracy | *M* | S: 0.91  D: 0.73 | S: 0.69  D: 0.74 | S: 0.80  D: 0.73 | A: 0.95  N: 0.86 | A: 0.95  N: 0.90 | A: 0.95  U: 0.88 | Sy: 0.87  Un: 0.7 |  |
|  | *SD* | S: 0.17  D: 0.25 | S: 0.25  D: 0.24 | S: 0.16  D: 0.21 | A: 0.11  N: 0.18 | A: 0.11  N: 0.16 | A: 0.11  U: 0.15 | Sy: 0.15  Un: 0.21 |  |
| Hits-FA | *M*  *SD* | 0.64  0.32 | 0.43  0.36 | 0.53  0.28 | 0.81  0.23 | 0.84  0.21 | 0.83  0.2 | 0.56  0.23 |  |
| D prime | *M* | 2.71 | 1.73 | 1.91 | 3.44 | 3.59 | 3.44 | 2.1 |  |
|  | *SD* | 1.51 | 1.60 | 1.30 | 1.25 | 1.18 | 1.16 | 1.03 |  |
| Response bias *c* | *M* | -0.45 | 0.08 | -0.12 | -0.22 | -0.14 | -0.22 | -0.37 |  |
|  | *SD* | 0.66 | 0.72 | 0.52 | 0.49 | 0.47 | 0.45 | 0.64 |  |
| S = same, D = different, M = mean, SD = standard deviation, Sy = synchronized, Un = unsynchronized, BbMAT-S = BbMAT-synchronized, BAT = beat alignment test, BBA = beat-based advantage. A = aligned, N = not aligned. | | | | | | | | | |

1.1.1. **Beat-based advantage task**

Paired-samples *t*-tests on *d’* showed that participants were more sensitive to simple (*M* = 2.71, *SD* = 1.51) compared to complex (*M* = 1.73, *SD* = 1.60) rhythms in the BBA, *t*(299) = 10.02, *p* < .001. There was also a difference in their response bias *c*, *t*(299) = 10.72, *p* < .001, with more negative response bias (i.e., more bias to respond same) in the simple (*M* = -0.45, *SD* = 0.66) compared to the complex (*M* = 0.08, *SD* = 0.72) condition. One-sample *t-*tests against 0 (no bias) show that there was a significant bias to respond “same” in the simple condition, *t*(299) = 11.81, *p* < .001, and a marginal bias to respond “different” in the complex condition, *t*(299) = 1.83, *p =* .068. See Figure S1.

**Figure S1**

*A) Sensitivity (d prime) and B) response bias c values for the simple and complex conditions in the beat-based advantage task (BBA). For response bias c, negative values show a bias to respond “same”, and positive values show a bias to respond “different”.*

1.1.2. **Beat Alignment Test**

Paired-samples *t*-tests showed that participants were significantly more sensitive to period errors (*M* = 3.59, *SD* = 1.18) compared to phase errors (*M* = 3.44, *SD* = 1.25), *t*(299) = 3.33, *p* = .001. Both period (*M* = -0.14, *SD* = 0.47, *t*(299) = 5.13, *p* < .001) and phase (*M* = -0.22, *SD* = .49, *t*(299) = 7.69, *p* < .001) conditions led to response biases inferior to 0, suggesting a bias to respond aligned. See Figure S2.

**Figure S2**

*A) Sensitivity (d prime) and B) response bias c values for the period and phase conditions in the beat alignment test (BAT). For response bias c, negative values show a bias to respond “aligned”, and positive values show a bias to respond “not aligned”.*

1.2. **Production Tasks**

Figure S3 shows the vector length R values and the inter-tap-interval for the production tasks, and Table S2 shows the means and standard deviations for the CV of ITI, vector length R, and the ITI for each task.

| **Table S2**  *Tapping Task Means (M) and Standard Deviations (SD) for the Coefficient of Variation of the Inter-tap-interval (CV of ITI), the vector length R, and the inter-tap-interval for Unpaced Tapping, Paced Tapping to Music 1, and Paced Tapping to Music 2* | | | |
| --- | --- | --- | --- |
| **Task** | **CV of ITI** | **Vector Length R** | **Inter-tap-interval** |
| Unpaced | *M* = 0.08, *SD* = 0.06 |  | *M* = 516.10, *SD* = 245.40 |
| Paced Music 1 | *M* = 0.13, *SD* = 0.12 | *M* = 0.73, *SD* = 0.29 | *M* = 470.95, *SD* = 181.50 |
| Paced Music 2 | *M* = 0.19, *SD* = 0.16 | *M* = 0.62, *SD* = 0.29 | *M* = 432.43, *SD* = 182.27 |

**Figure S3**

*A) Vector length R and B) inter-tap-interval (ms) for the paced tapping to music 1, paced tapping to music 2, and unpaced tapping (ITI only).*

*Note:* Dotted lines in B represent 600ms (the inter-beat-interval of the paced tapping stimuli) and 300ms, its subdivision.

**1.3 Comparison with Fiveash et al., 2022**

Figure S4 shows the distributions of all shared perception and production tasks in Fiveash et al. (2022) and the current study. Independent two-sample *t*-tests (Welch) showed that performance on the BAT was higher in the internet-based sample compared to the lab-based sample, *t*(37.93) = 3.21, *p* = .003, *d* = 0.58, whereas performance on the BBA was higher in the lab-based sample, *t*(38.32) = 2.62, *p* = .01, *d* = 0.47. There was no difference between the internet-based sample and the lab-based sample for the BbMAT, *t*(34.58) = 1.01, *p* = .32, *d* = 0.20. For production, the CV of ITI was higher (more variable) for the lab-based sample compared to the internet-based sample, *t*(29.6) = 2.67, *p* = .01, *d* = 0.66. There was no difference between the internet-based and lab-based samples for PM1, *t*(33.54) = 1.28, *p* = .21, *d* = 0.26 or PM2, *t*(29.13) = 0.29, *p* = .78, *d* = 0.06.

**Figure S4**

*A) Shared perception tasks from Fiveash et al., 2022 (n = 31) and the current study (n = 300). B) Shared production tasks from Fiveash et al., 2022 (n = 30, two data points missing for Paced Music 2), and the current study.*

**A)**

**B)**

2. **Principal Components Analysis**
   2.1 **Combined Perception + Production**

Three dimensions were reported and discussed in more detail in the main manuscript. In addition, Dimension 4 positively correlated with unpaced tapping and BBA, and negatively correlated with: BbMAT, paced tapping to music 2 (PM2), and BAT. This dimension was difficult to interpret. Dimension 5 correlated positively with BBA, PM2, BbMAT, and PM1. It correlated negatively with BAT and unpaced tapping. It is possible that this dimension was being driven by a distinction between paced and unpaced tapping; however, there were also perception variables contributing to this potential distinction too. See Figure S4 for Dimensions 4 and 5 compared to Dimension 1, and Table S2 for statistical values.

**Figure S5**

*Dimensions 4 and 5 compared to Dimension 1 emerging from the Principal Components Analysis for All Perception and Production Tasks.*

*Note*: BAT = Beat Alignment Test; BbMAT = Burgundy Best Musical Aptitude Test Synchronisation Task; BBA = Beat-Based Advantage task. Unpaced and paced tapping tasks were reversed so that higher scores reflected better (less variable) performance.

| **Table S3**  *Overview of the Dimensions, Variance Explained, and Contributing Factors of the Principal Components Analyses (PCA) for the Perception + Production PCA (Dimensions 4 and 5).* | | | | |
| --- | --- | --- | --- | --- |
| **PCA** | **Dim** | **Variance** | **Contributing Tasks** |  |
| Perception + Production | Dim 4 | 13.84% | UnP: *r*(296) = 0.78, *p* < .001  BBA: *r*(296) = 0.24, *p* < .001  *BbMAT: *r*(296) = -0.24, *p* < .001  *PM2: *r*(296) = -0.23, *p* < .001  *BAT: *r*(296) = -0.21, *p* < .001 |  |
|  | Dim 5 | 9.74% | BBA: *r*(296) = 0.35, *p* < .001  PM2: *r*(296) = 0.21, *p* < .001  BbMAT: *r*(296) = 0.14, *p* = .01  PM1: *r*(296) = 0.13, *p* = .03  *BAT: *r*(296) = -0.60, *p* < .001  *UnP: *r*(296) = -0.14, *p* = .01 |  |
| *Note:* *refers to negative contributions. Dimensions 1-3 are reported in the main text. Dim = Dimension; BAT = Beat Alignment Test; BbMAT = Burgundy Best Musical Aptitude Test Synchronisation Task; BBA = Beat-Based Advantage task; PM1 = Paced Tapping to Music 1; PM2 = Paced Tapping to Music 2; UnP = Unpaced Tapping. | | | | |

**2.2. Production Variables and Self-Report Production Measures**

An exploratory PCA was run to investigate how the two self-report measures: “I can tap in time with a musical beat” and “I am a good dancer; I dance in rhythm with the music” related to actual production measures. The PCA revealed five dimensions, accounting for 100% of the variance. Only the first three will be described, as they each accounted for more than 10% of the variance, whereas Dimension 4 (9.79%) and Dimension 5 (4.95%) accounted for less than 10% each. Dimension 1 (42.94%) correlated positively with all tests/responses: PM1, *r* = .84, *p* < .001; PM2, *r* = .83, *p* < .001; tap with beat, *r* = .59, *p* < .001; unpaced tapping, *r* = .49, *p* < .001; good dancer, *r* = .40, *p* < .001. Dimension 2 (26.18%) positively correlated with the self-report statements: good dancer, *r* = .78, *p* < .001; tap with beat, *r* = .63, *p* < .001, but correlated negatively with the production tasks: unpaced: *r* = -.35, *p* < .001; *r* = -.33, *p* < .001; PM2, *r* = -.27, *p* < .001; PM1. Dimension 3 (16.15%) correlated positively with unpaced tapping, *r* = .79, *p* < .001, and good dancer, *r* = .12, *p* = .03, but correlated negatively with PM2, *r* = -.35, *p* < .001 and PM1, *r* = -.21, *p* < .001. See Figure S5A for the dimension graphs.

Three clusters were revealed through the hierarchical cluster analysis (see Figure S5B). Cluster 1 (*n* = 54) showed negative loadings with the tap with beat response, *v* = -3.28, *p* = .001, as well as the tapping tasks: unpaced tapping, *v* = -6.79, *p* < .001; PM2, *v* = -11.79, *p* < .001; and PM1, *v* = -14.44, *p* < .001. These were participants who were weak at tapping and knew it. Cluster 2 (*n* = 137) showed positive loadings with PM1, *v* = 3.96, *p* < .001; and unpaced tapping, *v* = 2.78, *p* = .005; but negative loadings with good dancer, *v* = -10.48, *p* < .001 and tap with beat, *v* = -6.48, *p* < .001. This cluster appears to be good tappers, but they do not know it. Cluster 3 (*n* = 107) had positive loadings for both self-report measures: good dancer, *v* = 11.20, *p* < .001; tap with beat, *v* = 9.37, *p* < .001; as well as the production tasks: PM2, *v* = 7.70, *p* < .001; PM1, *v* = 7.48, *p* < .001; unpaced, *v* = 2.56, *p* = .01. These appear to be good tappers and they know it.

Years of musical training was predicted by cluster, *F*(2, 166.38) = 4.10, *p* = .02, ω² = 0.04, 95% CI [.00, .10], which appeared primarily driven by more musical training in Cluster 3 (*good tappers who know it*; *M* = 1.93, *SD* = 3.84, range = 0-25y) compared to Cluster 2 (*good tappers who do not know it*, *M* = 0.90, *SD =* 2.58*,* range *=* 0-15y), Est = 1.04, *p* = .046, *d* = 0.32, and Cluster 1 (*weak tappers who know it*, *M* = 0.69, *SD* = 1.78, range = 0-10y), Est = 1.25, *p* = .015, *d* = 0.42. There was no difference between Cluster 1 and 2, Est = 0.21, *p* = .79, *d* = 0.10.

**Figure S6**

*A) Principal Components Analysis for the Production Variables and the Self-Report Responses:* *I am a good dancer; I dance in rhythm with the music* *and* *I can tap in time with a musical beat. B) Hierarchical Cluster Analysis for the Clusters related to the Dimensions.*

3. **Multinomial Logistic Regression**

The multinomial logistic regression provides percentages in relation to the sensitivity of the classification (i.e., correct identification), the specificity (i.e., correct exclusion), and the precision (i.e., correct predictions). All values for each hierarchical cluster analysis are shown in Table S3.

| **Table S4**  *Multinomial Regression Classification Accuracy for Group Clusters* | | | |
| --- | --- | --- | --- |
| PCA | Sensitivity | Specificity | Precision |
| **Perception** |  |  |  |
| Cluster 1 | 99.11% | 100% | 100% |
| Cluster 2 | 100% | 99.48 | 99.09% |
| Cluster 3 | 98.73% | 99.54% | 98.73% |
| **Production** |  |  |  |
| Cluster 1 | 100% | 100% | 100% |
| Cluster 2 | 100% | 100% | 100% |
| Cluster 3 | 100% | 100% | 100% |
| **Perception + Production** |  |  |  |
| Cluster 1 | 96.61% | 99.58% | 98.28% |
| Cluster 2 | 98.20% | 98.40% | 97.32 |
| Cluster 3 | 99.22% | 99.41% | 99.22% |
| *Note*: PCA refers to the principal components analysis. | | | |

4. **Self-Evaluation of Music Skills and Task Performance**

To investigate whether participants’ ratings of different music-related statements related to performance on perception and production tasks, spearman correlations were run between the six tasks and the music-related statements: (1) “I can tap in time with a musical beat”*;* (2) “I am a good dancer; I dance in rhythm with the music”; (3) “I have trouble recognising songs without the lyrics”; (4) “I am rarely able to notice if someone is singing out of tune”. Correlations are reported in Table S4 and shown in Figure S6. Interestingly, after controlling for multiple comparisons (Holm-Bonferroni), the response to the statement “I can tap in time with a musical beat” correlated with both of the paced tapping tasks, the BAT, and the BBA. The statement “I am rarely able to notice if someone is singing out of tune” is a measure indicating potential amusia, and responses to this statement correlated negatively with the BAT, the BBA, and paced tapping to music 1.

| **Table S5**  *Spearman correlations between perception (d prime: BAT, BBA, BbMAT) and production (coefficient of variation of the inter-tap-interval: unpaced tapping, paced tapping to music 1, paced tapping to music 2) task performance and music-related self-report responses. Tapping measures have been reversed so that higher performance is better. See Figure S6 for correlational scatter plots.* | | | | |
| --- | --- | --- | --- | --- |
|  | Tap with beat | Good dancer | Trouble recognising songs | Rarely notice out of tune |
| BAT | *r* = .23, *p* < .001* | *r* = .18, *p* = .002* | *r* = -.11, *p* = .06 | *r* = -.31, *p* < .001* |
| BBA | *r* = .19, *p* = .001* | *r* = .03, *p* = .63 | *r* = -.04, *p* = .45 | *r* = -.19, *p* = .001* |
| BbMAT | *r* = .06, *p* = .27 | *r* = .06, *p* = .32 | *r* = -.06, *p* = .30 | *r* = -.10, *p* = .08 |
| Unpaced | *r* = .13, *p* = .03 | *r* = .03, *p* = .64 | *r* = -.01, *p* = .86 | *r* = -.05, *p* = .43 |
| PM1 | *r* = .34, *p* < .001* | *r* = .16, *p* = .005 | *r* = -.16, *p* = .006 | *r* = -.21, *p* < .001* |
| PM2 | *r* = .35, *p* < .001* | *r* = .16, *p* = .005 | *r* = -.13, *p* = .03 | *r* = -.16, *p* = .006 |
| Note: Tap with beat = “I can tap in time with a musical beat”. Good dancer = “I’m a good dancer; I dance in rhythm with the music”. Trouble recognising songs = “I have trouble recognising songs without the lyrics”; Rarely notice out of tune = “I am rarely able to notice if someone is singing out of tune”. All correlations had a sample size of 298 participants, as the two participants missing production data were removed. *refers to correlations that are still significant after Holm-Bonferroni correction for all 24 comparisons in the table. | | | | |

**Figure S7**

*Correlations for the four music-related self-report responses and performance on the perception (BAT, BbMAT, and BBA) and production (unpaced tapping, paced tapping to music 1, paced tapping to music 2) tasks. Regression lines with linear model fitted for illustration, shaded error bars represent one standard error of the mean, individual points represent individual participant responses. See Table S4 for detailed information.*
